# A methodological scoping review on EMG processing and synergy-based results in muscle synergy studies in Parkinson’s disease

**DOI:** 10.3389/fbioe.2024.1445447

**Published:** 2025-01-06

**Authors:** Valentina Lanzani, Cristina Brambilla, Alessandro Scano

**Affiliations:** Institute of Intelligent Industrial Systems and Technologies for Advanced Manufacturing (STIIMA), Italian Council of National Research (CNR), Milan, Italy

**Keywords:** Parkinson’s disease, muscle synergies, clinical scales, uncontrolled manifold, EMG, motor control, postural balance

## Abstract

**Introduction:**

Parkinson’s Disease is the second most common neurodegenerative disease in the world. It affects mainly people over 65 and the incidence increases with age. It is characterized by motor and non-motor symptoms and several clinical manifestations. The most evident symptom that affects all patients with Parkinson’s Disease is the impairment of motor control, including bradykinesia, tremor, joint rigidity, and postural instability. In the literature, it has been evaluated with muscle synergies, a well-known method for evaluating motor control at the muscular level. However, few studies are available and there is still a major gap to fill to exploit the potential of the method for assessing motor control in Parkinson’s Disease, both in the understanding of physiopathology and clinical practice.

**Methods:**

In the light of understanding and fostering future developments for the field, in this review we initially screened 212 papers on Scopus and Web of Science and selected 15 of them to summarize the main features of investigations that employed muscle synergies to analyze patients with Parkinson’s Disease. We detailed the features of the screened papers by reporting the clinical findings, a detailed report of EMG processing choices and synergy-based results.

**Results:**

We found that synergistic control is in general altered in patients with Parkinson’s Disease, but it can improve if patients are subjected to pharmacological and rehabilitation therapies. Moreover, a further understanding of synergistic control in Parkinson’s patients is needed.

**Discussion:**

We discuss the future developments in the field with a detailed assessment of the topic on the view of physicians, including the most promising lines of research for clinical practice and from the perspective of engineers, for methodological application of synergistic approaches.

## 1 Introduction

### 1.1 Parkinson’s disease

With a global prevalence of more than 6 million individuals, Parkinson’s disease (PD) is the second most common neurodegenerative disease in the world (Tolosa et al., 2021). According to the Global Burden of Disease study, PD is a neurological disorder which spreads so fast as to be compared to a pandemic disease (Dorsey et al., 2018). Parkinson spreading is fueled by aging populations, increasing longevity, and the by-products of industrialization; all these factors could lead the burden of PD to exceed 17 million affected people by 2040 (Dorsey et al., 2018). Parkinson affects mainly people over 65 and the incidence increases with age, even if also many individuals under 50 develop the disease (Dorsey et al., 2018). PD is characterized by different motor and non-motor symptoms which can affect patients in different manners because every person has their unique disease manifestation (Bloem et al., 2021). The lack of dopamine in the basal ganglia leads to classical Parkinsonian motor symptoms: bradykinesia, tremor, rigidity, and later postural instability. In particular, selective loss of dopaminergic neurons in the striatum causes impairment of motor control, changes in cerebellar activity and in the interaction between the basal ganglia and cerebellum, contributes to the onset of tremor, and the dysfunction of the basal ganglia output determines the abnormalities of balance and gait (Radhakrishnan and Goyal, 2018). These motor symptoms are often preceded by non-motor symptoms which may onset years before. Non-motor symptoms include sleep disorders, hyposmia, disturbance in autonomic function (e.g., orthostatic hypotension, urogenital dysfunction, and constipation), cognitive impairment, mood disorders, and pain. The administration of drugs, that act on dopaminergic transmission, is the most effective medical treatment to relieve motor symptoms in PD patients and Levodopa is considered the Gold Standard therapy, in spite of its long-term use seems to cause motor fluctuations and dyskinesias (Radhakrishnan and Goyal, 2018). Mechanisms underlying these motor complications are still unclear, so recent studies decided to apply an alternative approach to study human motor control analyzing muscle synergies trying to understand how motor control changes in PD patients compared to healthy people and how it changes when PD patients assume their medication (Park et al., 2014). Indeed, it is still unclear how PD affects postural strategies in terms of number of muscle synergies and how dopaminergic treatment changes postural control in PD patients (Mileti et al., 2020a). There are still many gaps that include how the central nervous system (CNS) controls the transition from the resting phase to the execution of voluntary movement that is influenced by the involuntary motor of tremor, how anticipatory postural adjustments (APAs) characteristics change in PD patients and how bradykinesia alters motor controls. In particular, during gait analysis, it is not clear if PD patients have more difficulty in initiating a step from wide stance because they are not able to increase muscle activation level for the lateral weight shift due to the bradykinesia or due to the failure to adapt APA motor programs (Rocchi et al., 2006).

### 1.2 Muscle synergies

To generate purposeful movement, the CNS has to coordinate many degrees of freedom of the musculoskeletal system, considering the nonlinear characteristics of the muscles and the dynamic interactions among the articulated segments of the body and between the body and the environment (D’Avella and Bizzi, 2005). Dynamic models were supposed to extract muscle activation until the early 2000s (Seth et al., 2018). However, this operation requires a very high computational effort. Thus, it was hypothesized that the control of movement is characterized by a simplified, low-dimensional strategy: the CNS activates movement building-blocks known as motor primitives and their combination allows to perform several complex motor patterns. This strategy allows for the efficient control of groups of neurons, motor-pools, and consequently muscles with low effort (Ó’Reilly and Delis, 2022). Several studies of the last two decades have demonstrated that motor primitives can be represented as muscle synergies which are usually defined as groups of coactive muscles with an invariant spatial structure that are flexibly recruited over time to transform movement goals into biomechanical outputs (Cheung and Seki, 2021; Allen et al., 2017). A small number of synergies could explain several muscle patterns and some synergies may be shared across different behaviors whereas others are task-specific (D’Avella and Bizzi, 2005). To determine the identity of the muscles belonging to a muscle synergy and the synergy’s time course of activation, dimensionality reduction algorithms were applied to analyze the multi-muscle electromyographic activities (EMGs). In a typical muscle synergy analysis, EMGs collected into a matrix **M** as column vectors are decomposed by an algorithm into two matrices: **W**, that represents the muscle synergies, and ***C***, which represents the synergies’ temporal coefficients. The most popular algorithm to extract muscle synergies is the non-negative matrix factorization (NMF). It extracts the statistical regularities from the EMG variability and represents these embedded data structures as muscle synergies (Cheung and Seki, 2021). The number of motor modules recruited to perform a motor-task is frequently considered as a measure of neuromuscular complexity, with higher complexity (i.e., more motor modules) associated with better motor performance (Allen et al., 2017). Muscle synergies exemplify the general idea that motor actions are composed of elementary building blocks that may be defined at different levels of the motor hierarchy (Cheung and Seki, 2021). The analysis of muscle synergies can be used to identify differences in neuromuscular control in both healthy and impaired populations during motor performance (Safavynia et al., 2011). Indeed, understanding general principles of neuromuscular control may help to improve patient screening to attribute the most appropriate rehabilitation pathway and guide the development of new interventions to enhance the reacquisition of movement skills lost through injury or disease (Allen et al., 2017). Thus, muscle synergies represent a non-optimal, yet still very parsimonious, approach to motor control.

### 1.3 The uncontrolled manifold method

Synergies can be extracted within a different framework based on the principle of motor abundance (Danna-Dos-Santos et al., 2007). The uncontrolled manifold (UCM) hypothesis assumes that the controller (the CNS) acts in a state space of control variables and selects in this space a manifold corresponding to a value or a time profile of a performance variable that needs to be stabilized (Danna-Dos-Santos et al., 2007). By doing this, the controller selectively limits the variability of control variables in a specific direction along which the selected performance variable changes while it allows higher variability in other directions (Krishnamoorthy et al., 2003). An example of performance variable is the center of pressure (COP) shift in the anterior-posterior direction, while the control variables are represented by muscle modes (M-modes, or synergies) (Falaki et al., 2017a). M-modes are defined as eigenvectors in the space of muscle activations using principal component analysis (PCA) with Varimax rotation and factor extraction. The original set of muscle variables is reduced into a smaller set of co-activating muscles considered as the control variables manipulated by CNS (Krishnamoorthy et al., 2003). Indeed, as in the NMF-based synergistic framework, muscles are not controlled by the CNS independently; moreover, it is not possible to associate changes in EMGs of individual muscles with changes in control variables, but it is necessary to identify a set of control variables which are presumably used by the CNS to control a large group of muscles for a set of tasks (Danna-Dos-Santos et al., 2007). The variance component within the UCM space (VUCM) has no effect on the performance variable, whereas the variance component within the space orthogonal to the UCM (VORT) does. The first variance leads to unchanged performance variable, while the second one leads to a change of performance variable. So, if VUCM is significantly higher than VORT, the system agrees with the UCM hypothesis and so most M-modes values variability leaves the value of the performance variable unaffected (Falaki et al., 2017b; Falaki et al., 2016). In our screening, we noted that standard synergies focus on how motor functionality can be eased by reducing the complexity of control, while the UCM method tries to analyze how variables critical for specific movements can be stabilized. Both models have the potential in unveiling the mechanisms of motor control in PD patients.

### 1.4 Study aims

As it is known in the literature (D’Avella et al., 2003), muscle synergies analysis is useful to understand how movement is controlled by the CNS at the neural level. Parkinson’s disease is characterized by motor disorders that are important to investigate from a clinical point of view to analyze the disease progressions and to find new and more efficacy rehabilitation therapy. There are several open points on the understanding of altered motor control in PD, intimately connected to the pathology and including APAs motor programs, bradykinesia, effects of brain stimulation, and the tremor influences on voluntary movements. These aspects are still unclear and they can impact on PD understanding and treatment under two points of view: clinical practice and research, and methodological implementation of synergies. Therefore, to better characterize the motor disorders of people with Parkinson’s disease and identify the most appropriate therapies, this review has two different aims. First, to assess and summarize in detail the clinical practice and research, as well as methodological choices made to analyze the EMG data and compute muscle synergies, in order to assess study reliability, inter-operability, and extract meaningful guidelines for future work; in this way there is an expansion of the previous review available for the field (e.g., Mileti et al., 2020b). Second, to summarize the findings of recent studies that have not been included in the available reviews, including studies that employed the UCM method for assessing motor control.

## 2 Literature search strategies and criteria

We reviewed studies in which muscle synergies were used to analyze the pathophysiology of people with Parkinson’s disease. The selected articles included only individuals affected by Parkinson’s disease and the studies that aimed at exploring disease-related issues through muscle synergies. We conducted a literature search using the following logical combination of keywords: (“muscle”) and (“synerg*”) and (“Parkinson”) and (“UCM”) in Scopus and Web of Science based on the Title, Abstract, and Keywords. The search included studies published until May 2024. A preliminary screening was conducted to exclude studies that did not involve muscle synergies and people with Parkinson’s disease, or the studies that involved only healthy participants based on the Abstract. Only journal papers in English were considered for screening. The complete query was:

TITLE-ABS-KEY (((muscle AND synerg*) OR UCM) AND Parkinson).

Then, duplicate articles and reviews were not considered in our research. Finally, we excluded all the articles that used non-EMG signals for synergistic analysis. Afterward, we extracted clinical information (e.g., number and features of participants, followed therapy, number, and type of muscles analyzed), the experimental protocols (e.g., the aim of the study, the study design and tasks), the data processing methods (e.g., muscle synergy models, synergy extraction methods and signal processing methods), and the main findings from the selected studies to provide a comprehensive summary.

## 3 Results

**FIGURE 1:**
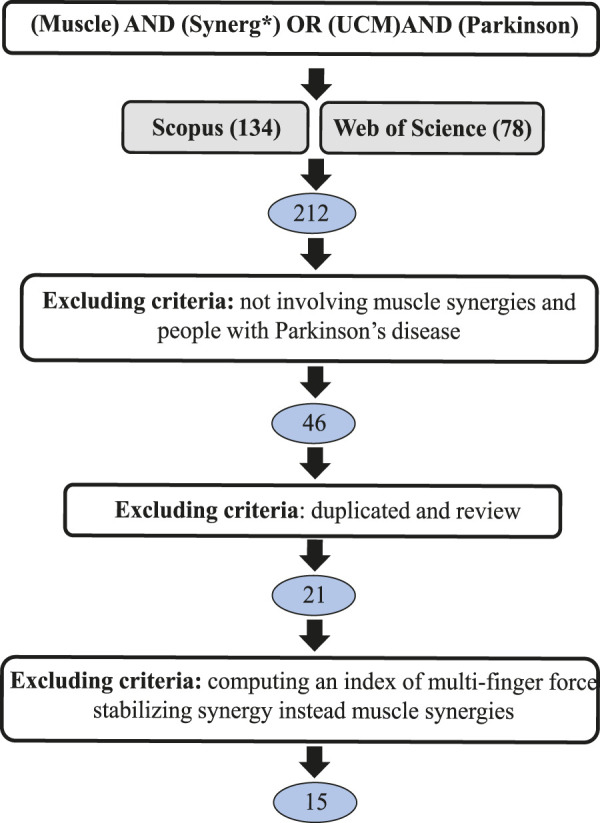
PRISMA diagram for literature review.

The PRISMA graph for our review is reported in Figure 1. According to our literature search criteria, a total of 212 papers were found in our screening in Scopus and Web of Science. The studies that did not satisfy the additional selection criteria were excluded. Finally, 15 papers were considered in this review. We organized our screening into four sections, highlighting different aspects as shown in Table 1.

**TABLE 1:** This review is divided into four sections to highlight relevant aspects of the screened studies.

Clinical characteristics of the studies (Table 2)	Processing of the Synergistic Variables (Table 3)	Synergy-based Results (Table 4)	Results in the framework of the UCM hypothesis (Table 5)
Aim of the study	Type and Number of tasks performed	Number of extracted synergies	M-modes
The study design	Number of experimental conditions	Spatial synergies variation (ΔW)	VUCM and VORT
Number of patients and case controls	Trials used to extract synergies	Activation coefficient variation (ΔC)	Synergies indices (ΔVz)
The scale of disability (H&Y)	Data filtering techniques		Anticipatory synergy adjustments
Anatomical segment	Data stacking techniques		
Drug therapy	Data normalization		
Therapy applied/exercise performed	Algorithm of synergies extraction		
Duration of therapy	Selection criteria of the Number of synergies		
Type of muscles			
Number of muscles			

### 3.1 Clinical characteristics of the studies

In Table 2, the clinical characteristics of the included studies are reported in detail.

**TABLE 2:** Clinical characteristics of the screened studies.

First author	Year	Aim of the study	The study design	N° of patients and case controls	Scale of disability H&Y	Anatomical segment	Type of therapy	Drug therapy	Duration of therapy	Type of muscles	Number of muscles
Ghislieri et al.	2023	To assess motor control changes in PD patients after bilateral DBS, based on a novel muscle synergy evaluation approach	Longitudinal study that compares patients before surgery, after 3 months, and after 1 year, and with healthy subjects	20 PD patients and 20 age-matched healthy controls	Before surgery = II-III stage; After surgery = I-II stage	Trunk and lower limbs	—	ON + patients were subjected to DBS surgery	—	LD, TFL, GMD, RF, LH and MH, VM, GL, PL, SOL, TA	12 muscles (most affected side/dominant side)
Falaki et al.	2023	To investigate changes in indices of muscle synergies prior to gait initiation and the effects of gaze shift in PD patients	Single-session comparison of patients with age-matched and healthy younger controls	10 PD patients 10 age-matched controls 9 young controls.	≤II stage	Lower limbs	Levodopa equivalent daily dose	ON	—	TA, SOL, GM, BF, RF, VL, TFL, ESL	16 bilateral channels (8 on the right and 8 on the left)
Bai et al.	2021	To investigate the change of synergy patterns from resting tremor to subsequent compound movement with voluntary reaching movement and tremor	Single-session comparison between PD patients and healthy controls	3 PD patients 8 healthy subjects.	—	Upper limbs	—	—	—	PC, DP, Bic, Tri, FCR, ECR	6 muscles mono-lateral
Freitas et al.	2020	To disambiguate the effects of levodopa from the impact of the disease on indices of postural stability in PD.	Single-session comparison between PD patients not subjected to Levodopa and healthy subjects, and the same patients with Levodopa	11 levodopa-naïve PD patients 11 healthy controls.	I-II stage for 10 patients and III- stage only for a patient	Lower limbs	Carbidopa/Levodopa	At the first trial is OFF then ON	Before and 60 min after the first dose of carbidopa/levodopa	RA, EST, ESL, TFL, VM, VL, RF, ST, BF, GL, GM, SOL, TA	13 muscles mono-lateral on the right side of the body
Mileti et al.	2020	To obtain a deeper insight into pathophysiological mechanisms associated with balance disorders in PD patients, under and not under dopaminergic treatment	Single-session comparison between PD patients subjected to Levodopa and healthy subjects, and with the same patients after a 12-h washout period from the drug	10 PD patients 10 healthy adults.	4 patients: I-II stage 5 patients: II-III stage 1 patient: III stage	Upper limbs	Levodopa	First Levodopa OFF Second Levodopa ON	Patients assume Levodopa regularly in the morning	PM, DP, Bic, Tri, EO, IL	12 muscles, 6 on the right side and 6 on the left side
Hu et al.	2019	To investigate the changes of muscle activation during resting tremor and voluntary movements evoked by cutaneous stimulation	Single-session comparison between patients with and without cutaneous stimulation	3 patients with tremor dominant symptom	—	Upper limbs	—	—	—	PM, DP, Bic, Tri, FCR, ECR	6 muscles mono-lateral on the tremor dominant hand
Hu et al.	2019	To evaluate how evoked cutaneous afferents impact the performance of voluntary movements in PD subjects when tremor is inhibited	Single-session study assessing the impact of evoked cutaneous afferents in patients and controls	10 patients with tremor 8 age-matched controls	II-III stage	Upper limbs	—	ON. Patients had medication 3h before the analysis	—	PM, DP, Bic, Tri, FCR, ECR	6 muscles from the right side of the limbs
Falaki et al.	2018	To explore the effects of DBS in PD patients on the synergic control of muscles in a task involving vertical posture	Single-session comparison between case controls, patients with and without DBS stimulation	10 PD patients 16 controls (8 for hand task, 8 for postural task)	> II stage	Lower limbs	DBS therapy	ON	—	TA, SOL, GM, GL, BF, ST, RF, VL, VM, TFL, ESL, EST, RA	13 of leg and trunk muscles on the right side of the body
Allen et al.	2017	To demonstrate changes in neuromuscular control of gait and balance in PD patients after short-term, dance-based rehabilitation	Longitudinal comparison between patients before and after the Adapted Tango rehabilitation intervention	6 patients.	II-III stage	Lower limbs	Their medication. Dance rehabilitation	ON	3 weeks of dance rehabilitation	RA, EO, EST, GMD, TFL, BF, RF, VM, GM, GL, SOL, PL, TA	13 muscles of the right-side leg and lower back
Falaki et al.	2017	To explore posture-stabilizing multi-muscle synergies in PD patients with (1) Analysis of inter-cycle variance; and (Dorsey et al., 2018) Analysis of motor equivalence	Two single-session studies. One analysis compared early-stage PD patients and controls The second analysis explored the effects of dopaminergic medication	First experiment: 11 PD patients 11 healthy adults Second experiment: 10 PD patients.	First: II stage Second: II-III stage	Lower limbs	—	First: ON drug condition Second: OFF-drug and ON drug condition	—	TA, SOL, GM, GL, BF, ST, RF, VL, VM, TFL, ESL, EST, RA	13 leg and trunk muscles on the right side of the body
Falaki et al.	2017	To explore the effects of dopamine-replacement drugs on multi-muscle synergies and their adjustments prior to a self-triggered perturbation in PD patients	Single-session study where PD patients were tested in OFF-DRUG and ON-DRUG conditions	10 patients	II-III stage	Lower limbs	Dopaminergic medication.	OFF-drug and ON-drug conditions	Patients took their medication regularly in the morning	TA, SOL, GM, GL, BF, ST, RF, VL, VM, TFL, ESL, EST, RA	13 surface muscles of the right side of the body
Falaki et al.	2015	To test that synergy indices during quiet standing and synergy adjustments to self-triggered postural perturbations would be reduced in PD patients	Single-session study where PD patients and controls were compared to assess the postural stability control	11 PD patients 11 controls.	≤ II stage, so without clinically identifiable postural instability	Lower limbs	—	ON	—	TA, SOL, GM, GL, BF, ST, RF, VL, VM, TFL, ESL, EST, RA	13 muscles surface muscles of the right side of the body
Rodriguez et al.	2013	To investigate motor modules and activation profiles during gait in PD patients and healthy older adults, and to investigate relationships between motor modules and biomechanical gait characteristics	Single-session study where PD patients and neurologically healthy older adults were compared	15 PD patients 14 age-matched healthy controls	—	Lower limbs	—	ON	—	SOL, GAS, TA, VM, RF, SM, BF, GM	8 bilateral leg muscles
Roemmich. et al.	2014	To investigate the effects of dopaminergic therapy on neuromuscular complexity during gait when PD patients are OFF meds and ON meds	Single-session study to compare the same group of PD patients having withdrawn from dopaminergic medication for at least 12 h and when optimally medicated	9 persons with mild-to-moderate PD.	—	Lower limbs	Orally-administered carbidopa/levodopa therapy	DRUG-OFF (withdrawn from dopaminergic medication for at least 12) DRUG-ON	—	SOL, GAS, TA, VM, RF, SM, BF, GM	8 bilateral leg muscles
Thenaisie et al.	2022	To uncover the principles through which the subthalamic nucleus encodes functional and dysfunctional walking in PD people	Single-session study in which PD patients were assessed during walking with DBS leads	18 PD patients.	—	Lower limbs	DBS leads in the left and right STN	OFF	—	TA, GM, GL, VL, ST, RF	6 leg muscles (bilateral, 12 total)

#### 3.1.1 Aim of the study

Muscle synergies were applied with several aims, including: 1) the investigation of postural control and how it changes prior to gait initiation; 2) the analysis of how voluntary movement is affected by tremor; 3) the evaluation of the difference between healthy controls and PD patients during waking on a treadmill; 4) the comprehension of pathophysiological mechanisms behind the Parkinson’s disease; 5) the assessment of motor control changes in PD after rehabilitation therapy.

##### 3.1.1.1 Investigation of postural control and how it changes prior to gait initiation

Falaki et al. investigated the effect of Parkinson’s disease on synergy indices during postural control and how they changed prior to gait initiation, exploring the posture-stabilizing multi-muscle synergies and synergy adjustments to self-triggered postural perturbations both in healthy subjects and in patients (Falaki et al., 2023; Falaki et al., 2017a; Falaki et al., 2016). Also, the influence of dopaminergic medication (Falaki et al., 2017b; Freitas et al., 2020) and the effect of deep brain stimulation (DBS) (Falaki et al., 2018) on the synergistic control of muscles in tasks involving vertical posture and in controlling anticipatory synergy adjustment were studied.

##### 3.1.1.2 Analysis of how voluntary movement is affected by tremor

Bai et al. and Hu et al. explored how voluntary movement was affected by tremor analyzing the change in muscle activation and synergy patterns. In particular, Hu et al. investigated the role of cutaneous stimulation assessing if it could change muscle activation both during resting tremor and voluntary movement, and observed how the cutaneous stimulation impacted on voluntary movements of PD patients when tremor was inhibited (Bai et al., 2021; Hu Z. et al., 2019; Hu Z.-X. et al., 2019).

##### 3.1.1.3 Evaluation of difference between healthy controls and PD patients during walking on a treadmill

Rodriguez et al. and Roemmich et al. evaluated subjects who walked on an instrumented treadmill with a preferred speed. Rodriguez et al. compared patients with Parkinson’s and healthy subjects to assess if they used a similar set of motor modules during gait and how synergistic parameters changed between the two groups. They also investigated the relationship between motor modules and biomechanical gait characteristics in patients and healthy controls (Rodriguez et al., 2013). Roemmich et al. analyzed the effects of dopaminergic therapy on neuromuscular complexity, by comparing the number, structure, and timing of lower extremity motor modules during gait when people with PD are withdrawn from dopaminergic therapy and when optimally-medicated (Roemmich et al., 2014).

##### 3.1.1.4 Comprehension of pathophysiological mechanisms behind Parkinson’s disease

Mileti et al. and Thenaisie et al. tried to understand the pathophysiological mechanisms behind Parkinson’s disease, such as how muscle synergies could highlight balance disorders and how the subthalamic nucleus could encode functional and dysfunctional walking in patients affected by Parkinson (Mileti et al., 2020a; Thenaisie et al., 2022).

##### 3.1.1.5 Assessment of motor control changes in PD after a rehabilitation therapy

Ghislieri et al. and Allen et al. conducted two longitudinal studies to assess motor control changes in walking tasks in individuals with Parkinson’s disease. In the first study, patients with Parkinson’s were subjected to bilateral DBS of the subthalamic nucleus and they were analyzed at three times: before surgery, after 3 months from the surgery, and after 12 months. During these sessions, muscle synergies were analyzed to evaluate how they changed and if they became similar to muscle synergy indices of healthy subjects (Ghislieri et al., 2023). Allen et al. demonstrated changes in neuromuscular control of gait in individuals with Parkinson’s disease after dance-based rehabilitation (Allen et al., 2017).

#### 3.1.2 Study design

**FIGURE 2:**
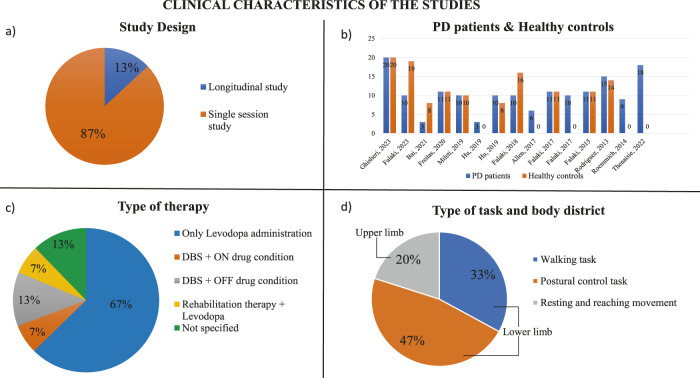
Clinical characteristics of the studies. **(A)** Study design (% on the number of screened papers). **(B)** Number of PD patients and healthy controls evaluated in the screened studies. **(C)** Type of task analyzed in the screened studies and anatomical body segment used to performed the specific task. **(D)** Type of therapies applied on PD patients to try to reduce their motor symptoms.

Two main types of studies were considered: single session and longitudinal (Figure 2A). Single session studies compared two experimental conditions (Mileti et al., 2020a; Falaki et al., 2023), while longitudinal studies followed a rehabilitation course or therapy in multiple sessions. Single session studies were mainly used to evaluate how muscle synergies change during postural control and gait initiation comparing patients with Parkinson’s disease who assumed their daily medication and patients with Parkinson who withdrew from their dopaminergic medication (Mileti et al., 2020a; Falaki et al., 2017a; Falaki et al., 2017b; Freitas et al., 2020; Roemmich et al., 2014). Moreover, single session studies were also applied to compare healthy controls and PD patients to underline the differences between their muscle synergies during a set of performed tasks (e.g., Rodriguez et al., 2013 and Falaki et al., 2016). On the contrary, only two longitudinal studies assessed the efficacy of a rehabilitation therapy in restoring patients’ muscle synergies (Allen et al., 2017; Ghislieri et al., 2023).

#### 3.1.3 Patients and case controls

The majority of the studies compared individuals with Parkinson’s disease and healthy subjects (Figure 2B). Usually, the number of patients and case controls was similar or around 10 (Mileti et al., 2020a; Falaki et al., 2017a; Falaki et al., 2016; Freitas et al., 2020; Hu Z. et al., 2019). The study conducted by Falaki et al. compared 10 PD patients with 10 healthy age- and sex-matched controls and then with 9 healthy young controls (Falaki et al., 2023). Only Ghislieri et al. and Rodriguez et al. considered a higher number of PD patients and healthy subjects analyzing 20 PD patients and 20 case controls and 15 PD patients and 14 healthy subjects respectively (Rodriguez et al., 2013; Ghislieri et al., 2023). Other two studies showed a bigger number of case controls than the number of patients with Parkinson’s disease, such as Falaki et al. who considered 10 PD patients and 16 healthy controls, and Bai et al. who examined 3 PD patients and 8 case controls (Falaki et al., 2018; Bai et al., 2021). Some studies included only individuals with Parkinson’s disease to compare different experimental conditions or to analyze specific features of pathology (Allen et al., 2017; Falaki et al., 2017b; Hu Z.-X. et al., 2019; Roemmich et al., 2014; Thenaisie et al., 2022). In most of the studies, the patients’ level of disability was evaluated through the Hoehn and Yahr scale (H&Y) that assesses the clinical stage of the patients with Parkinson’s disease (Hoehn and Yahr, 1967). All studies of our screening considered patients at the I-II stage or at the II-III stage. In these stages, the disease affects both sides of the body, but it does not compromise the postural balance except to a small degree that still allows for independent daily living.

#### 3.1.4 Anatomical segment, type, and number of muscles

Eleven studies in our screening investigated muscles of the lower limbs; in fact, they evaluated walking tasks (Allen et al., 2017; Rodriguez et al., 2013; Roemmich et al., 2014; Thenaisie et al., 2022; Ghislieri et al., 2023), the maintenance of vertical posture (Falaki et al., 2017a; Falaki et al., 2017b; Falaki et al., 2016; Falaki et al., 2023; Freitas et al., 2020; Falaki et al., 2018) or balance control (Mileti et al., 2020a; Allen et al., 2017). In the case of lower limb analysis, the muscles considered were rectus abdominis (RA), thoracic erector spinae (EST), lumbar erector spinae (ESL), tensor fasciae latae (TFL), vastus medialis (VM), vastus lateralis (VL), rectus femoris (RF), semitendinosus (ST), biceps femoris (BF), gastrocnemius lateralis (GL), gastrocnemius medialis (GM), soleus (SOL), and tibialis anterior (TA), which were involved in the tasks analyzed. Most of the studies evaluated 12–13 muscles considering only the right side of the body (Allen et al., 2017; Falaki et al., 2017a; Falaki et al., 2017b; Falaki et al., 2016; Freitas et al., 2020; Falaki et al., 2018), whereas Ghislieri et al. analyzed the most affected side of PD patients and the dominant side of the healthy participants (Ghislieri et al., 2023). Instead, other studies considered 6-8 bilateral leg muscles investigating the movement of both subjects’ legs while walking (Mileti et al., 2020a; Falaki et al., 2023; Rodriguez et al., 2013; Roemmich et al., 2014; Thenaisie et al., 2022). The upper limb functionality was investigated in four studies where reaching movements were evaluated (Mileti et al., 2020a; Bai et al., 2021; Hu Z. et al., 2019; Hu Z.-X. et al., 2019). Three studies analyzed 6 monolateral muscles of the upper limb, (Bai et al., 2021; Hu Z. et al., 2019; Hu Z.-X. et al., 2019), while Mileti et al. investigated 12 upper body muscles considering both sides (Mileti et al., 2020a). The upper limb muscles considered in these four studies were the clavicular head of pectoralis major (PM), deltoid posterior (DP), biceps brachii (Bic), triceps brachii (Tri), flexor carpi radialis (FCR), and extensor carpi radialis (ECR). The distribution of anatomical segments and muscles is shown in Figure 2D.

#### 3.1.5 Therapies

Five studies considered PD patients who assumed their daily medication (Allen et al., 2017; Falaki et al., 2016; Falaki et al., 2023; Hu Z. et al., 2019; Rodriguez et al., 2013). The gold standard therapy used today is the oral-administration of dopaminergic drugs, such as Levodopa or Carbidopa (Radhakrishnan and Goyal, 2018) (Figure 2C). In addition to typical drug therapy, one study applied a rehabilitation therapy which consisted of 3 weeks of dance rehabilitation, specifically adapted tango lessons (Allen et al., 2017).

In the screened studies, another solution to try to reduce debilitating motor symptoms of PD patients, such as tremor and dystonia, was the application of DBS, which is a surgical treatment characterized by the implantation of leads in the areas of the brain deputed to control movement. This type of treatment could be applied alone or in addition to the pharmacological therapy. This surgical procedure was applied in three studies (Falaki et al., 2018; Thenaisie et al., 2022; Ghislieri et al., 2023).

The remaining studies considered PD patients in two different conditions: first, they tested PD patients who had a withdrawn from their dopaminergic medication for at least 12 h (OFF-drug condition), and then they analyzed the same PD patients an hour after taking their daily drug (Mileti et al., 2020a; Falaki et al., 2017a; Falaki et al., 2017b; Freitas et al., 2020; Roemmich et al., 2014). In two studies the type of therapy was not specified (Bai et al., 2021; Hu Z.-X. et al., 2019).

### 3.2 Processing Synergistic variables

Processing Synergistic variables are summarized in Table 3.

**TABLE 3:** Synergy data processing.

First author	Year	Type and N of tasks	Experimental conditions	Trials used to extract synergies	Data filtering technique	Data stacking technique	Data normalization	Extraction algorithm	Selection criteria of the N° of synergies
Ghislieri et al.	2023	Walking barefoot, at self-selected speed, for 5 min back and forth over a 9-m straight walkway	Subjects with PD were evaluated during locomotion at three time points (before surgery, after 3 and 12 months), while the control group performed the overground walking task only once	Only gait cycles belonging to the straight path were considered. In the study muscle synergies were extracted from each 10-gait-cycle time epoch	band-pass filter (10–500 Hz, 2nd IIR Butterworth), high-pass filter (35 Hz, 8th order IIR Butterworth), rectification, low-pass filter (12 Hz, 4th order IIR Butterworth)	sEMG envelopes were grouped into time epochs of 10 concatenated gait cycles. Then time-normalized to 1,000 samples per gait cycle	EMG envelope was amplitude-normalized to the global maximum of each acquired muscle for each participant	NMF	“elbow” criterion (such as 90% of the fraction of data variation accounted for by the muscle synergy model)
Falaki et al.	2023	3 tasks: quiet standing (QS), continuous voluntary sway (VS), and gait initiation	Participants stood and leaned forward to shift their COP AP 3 cm anterior to the initial position for about 2–3 s, and initiate gait in a self-paced manner by making two steps (right leg). This gait initiation was performed with a quick prior turn of the head (Turn) and without it (no-Turn)	The QS task was used to measure the baseline EMG activity. VS_AP and VS_ML data were used to define a low-dimensional set of muscle groups with co-varied changes in their activity. Only sway cycles during the middle 25 s of each VS trial were accepted	band-pass filter (20–350 Hz, 4th-order Butterworth), rectification, 100-ms moving average window. An electromechanical delay of 50 ms was applied. EMG and COP data from the VS task were averaged over 50-ms windows	Not specified	EMG signals were normalized by subtracting QS background activity and dividing by the muscle’s peak activation during VS trials	PCA	Kaiser criterion in addition to visual inspection of the scree plots. Each M-mode contained at least one significantly loaded muscle, i.e., a muscle with a loading factor beyond ±0.5
Bai et al.	2021	Relax at the initial point; voluntary forward reaching movement as fast as possible	Subjects sat in front of a platform with their hand placed on an arm support apparatus. To perform the reaching movement a target point was situated 24 cm away	“tremor prior”: EMGs of distinct tremor cycles between −4 and −2 (s) prior to the “go”; “tremor after”: tremor EMGs between 2 and 4 (s) after compound movement; “compound movement”: EMG of forward reaching movement between 0 and 2 (s) after the “go” cue	Notch-filter (50 Hz and 120 Hz), band-pass filter (20–390 Hz), rectification, low-pass filter (20Hz)	The EMGs of tremor cycles and the EMG of forward reaching movements were concatenated in distinct matrices	No normalization was performed	NMF	The subjects’ average VAF exceeds 90%
Freitas et al.	2020	Three postural tasks: quiet standing (QS), voluntary whole-body sway (VS), and load release (LR)	During QS and VS, participants were asked to keep their arms crossed over the chest. In the LR they stayed in the same position as in the previous trial, but with their arms extended forward and palms facing upward while holding a load	The EMG data obtained from QS trial were used during offline data processing. The data from VS trials were later used to identify the jointly activated muscle group	Band-pass filter (20–350 Hz, 4th-order Butterworth), rectification, integration over 50-ms time windows from each cycle	Data were concatenated across the sway cycles analyzed	EMG signals were normalized by subtracting QS background activity and dividing by the muscle’s peak activation during VS trials	PCA with Varimax rotation and factor extraction	To contain at least one muscle with a significant loading (absolute magnitude >0.5)
Mileti et al.	2020	Two perturbation tasks with robotic platform: (i) Low (L), and (ii) High (H) frequency	Participants were asked to stand in their comfortable stance on top of the robotic platform with their feet symmetrically placed on the center of the platform. Tasks lasted 8s and 6s respectively for the L and H conditions	All tasks were repeated three times in a random order	High-pass filter (35 Hz, 5th-order Butterworth), notch filter (50 Hz), rectification, low-pass filter (5 Hz). All signals were resampled to 100 samples	For each condition, the average activation signal between three repetitions was computed	EMG was normalized to the maximum activation amplitude across all sessions for each muscle	NMF	Total VAF ≥90% local VAF ≥75%
Hu et al.	2019	2 tasks: resting and reaching task	PD patients were seated in front of a table with the tremor dominant hand on the horizontal plane covered by a cover plane. After relaxing, patients had to do fast-reaching movements between the horizontal plane and the cover plane using the tremor arm without visual feedback	The length of each tremor cycle was about 550 samples (about 0.23 s). For each synergy extraction, 2 s duration of EMG signals before and during electrical stimulation (without and with stimulation duration) were selected for analysis	Notch-filter (50 and 120 Hz), band-pass filter (20–390 Hz), rectification, low-pass filter (20 Hz)	To reconstruct the EMG matrix of all the trials for synergy analysis, the averaged tremor group of each trial was extracted	No normalization was performed	NMF	The VAF of more than half of the patients exceeds 80%
Hu et al.	2019	Frontal reaching movement.	Patients performed targeted reaching movements without visual feedback of their hands, with random on/off cutaneous stimulation applied unknowingly. Both patients and controls, seated at a table with their hand supported to cover their upper limb, performed the task	For the synergy extraction of tremor, one tremor cycle of six muscle EMGs was extracted. The length of each tremor cycle was about 550 samples (0.23 s). 2s duration points of EMG signals before and during stimulation were selected for the analysis	Notch filter (50 Hz and 120 Hz), band-pass filter (20–390 Hz), rectification, low-pass filter (20 Hz)	The tremor group was averaged by all the tremor groups of that trial	No normalization was performed	NMF	80% level of VAF
Falaki et al.	2018	Three main tasks: quiet standing (QS), continuous voluntary sway (VS), and load release (LR)	Participants stood barefoot on a force plate and performed hand and postural tasks twice (DBS-ON and DBS-OFF) with 10-minute breaks. They held a load with extended arms, leaned forward to shift the COP AP 3 cm anteriorly, maintained the posture for 2–3 s, and then released the load with a quick bilateral arm abduction	Subjects repeated this task for 24 trials with 10-s rest between trials and a 2–3 min break after each set of 12 trials	Band-pass filter (20–350 Hz, 4th-order Butterworth), rectified, and low-pass filter (100-ms moving average window)	Not specified	EMG signals were normalized by subtracting QS background activity and dividing by the muscle’s peak activation during VS trials	PCA with Varimax rotation with factor extraction	Based on the Kaiser criterion, the first four PCs with the greatest eigenvalues were selected as M-modes after applying PCA with Varimax rotation and factor extraction on the correlation matrix of the averaged EMGs
Allen et al.	2017	Walking task and reactive balance assessments	During walking assessments, each participant walked overground at a self-selected walking speed for ∼7.5 m. During reactive balance assessments, postural responses to ramp-and-hold translations of the support surface during standing were recorded while participants stood on an instrumented platform that translated in 12 directions in the horizontal plane	For walking, at least five total gait cycles per walking condition were included in the analyses. For reactive balance, EMG data were analyzed during four different time bins: one before the perturbation and three during the automatic postural response	High-pass filter (35 Hz), demeaning, rectification, low-pass filter (40 Hz)	For the walking, trials were concatenated. For the balance, mean muscle activity values for each muscle during each trial were used	EMG data matrices were normalized to the maximum activation observed during walking	NMF	The number of motor modules was chosen such that the lower bound of the 95% confidence interval (CI) on VAF exceeded 90%
Falaki et al.	2017	Two tasks: Quiet standing (QS) and Voluntary sway (VS)	In the QS task, participants stood on a force platform for 30 s. In the VS task, they performed continuous AP sways about the ankle, shifting COP_AP between two screen targets while minimizing COP_ML deviations	For the VS trial, the data within {3 s; 28 s} time interval was accepted. The time interval between two consecutive anterior-most COP_AP coordinates was defined as a sway cycle. On average, each participant performed 10 cycles within each VS trial	Band-pass filtered (20–350 Hz, 4th-order Butterworth), rectification, 100-ms window moving average filter	Trial-by-trial analysis	EMG signals were normalized by subtracting QS background activity and dividing by the muscle’s peak activation during VS trials	PCA with Varimax rotation with factor extraction	Kaiser criterion in addition to visual inspection of the scree plots. Each M-mode contained at least one significantly loaded muscle, i.e., a muscle with a loading factor beyond ±0.5
Falaki et al.	2017	Four tasks: Quiet standing (QS), Voluntary sway (VS), Load release (LR), and Fast-body motion (FBM)	Subjects stood on a force platform with their feet parallel and shoulder width apart and the platform recorded components of the forces applied to the surface of the platform along the AP direction (FX) and the vertical direction (FZ), and the moment of force around the Y-axis	For VS trials only the data within the interval {3 s; 28 s} were used. On average, each subject performed 10 full cycles within this interval	Band-pass filter (20–350 Hz, 4th-order Butterworth), rectification, low-pass filter (moving average 100-ms window). To quantify ASAs, the synergy index was averaged within two-time intervals during the LR task	Not specified	EMG signals were normalized by subtracting QS background activity and dividing by the muscle’s peak activation during VS trials	PCA with factor extraction after Varimax rotation	Based on the Kaiser criterion, four PCs were accepted as M-modes, confirmed by visual inspection of the scree plot
Falaki et al.	2015	Three tasks: voluntary sway (VS), fast-sway (FS), and load release (LR)	To ensure participant safety, all subjects used a safety harness. Initially, subjects were asked to stand on the force plate while keeping their feet parallel at shoulder width apart; the foot position was marked and reproduced across trials	For the VS task, signals in the interval {3 s; 28 s} were considered. On average, each participant performed ten full cycles within this period. During the FS and LR tasks, trials without major errors were accepted, 17 for both groups	Band-pass filter (20–350 Hz, 4th-order Butterworth), rectification, low-pass filter (moving average 100-ms window). To quantify ASAs, the synergy index was averaged within two-time intervals during the LR task	Not specified	EMG signals were normalized by subtracting QS background activity and dividing by the muscle’s peak activation during VS trials	PCA with factor extraction after Varimax rotation	Based on the Kaiser criterion, four PCs were accepted as M-modes, confirmed by visual inspection of the scree plot
Rodriguez et al.	2013	1 task: walking on an instrumented treadmill	Subjects walked for 10 min on an instrumented treadmill at their preferred speed	The EMG, kinematic, and kinetic data were collected over the last 4 min of the walking	High-pass filter (35 Hz, 4th-order Butterworth), demeaning, rectification, low-pass filter (7 Hz, 4th-order Butterworth)	Trial-by-trial analysis: Each gait cycle was analyzed separately assuming that muscle weightings were fixed for that cycle while activation profiles were allowed to vary across gait cycles	EMG signal from each muscle was normalized to its peak value. Each EMG signal was also normalized temporally to 0%–100% of the gait cycle	NMF	95% VAF of all muscles combined
Roemmich et al.	2014	2 tasks: overground walking and walking on a treadmill at a preferred speed	Patients walked overground and then walked on the treadmill for 5 min on the DRUG-OFF condition. An 1h later assuming their daily medication, they walked with the same preferred speed. Then, participants also performed ten overground gait trials at a self-selected comfortable pace while ON meds	Ten overground gait trials at a self-selected pace (OFF meds OG) and on the treadmill for 1 min at 1.0 m/s (OFF meds Fast). They then walked on the treadmill at their preferred walking speed (OFF meds Pref) for 5 min. Then, on the treadmill at the same speed previously selected while OFF meds (ON meds Pref)	High-pass filter (35 Hz, 4th-order Butterworth), demeaning, rectification, low-pass filter (7 Hz, 4th-order Butterworth)	Trial-by-trial analysis: Each gait cycle was analyzed separately assuming that muscle weightings were fixed for that cycle while activation profiles were allowed to vary across gait cycles	The amplitude of each EMG signal was normalized to its peak value during the trial and each signal was time-normalized to 100% of the gait cycle	NMF	the total %VAF across all reconstructions collectively reached 95%
Thenaisie et al.	2022	2 tasks: walking and obstacle task	Walking tasks (small and big steps): Patients stood for 3 s, walked at a comfortable speed along marked lines, stopped for 3 s at the end, performed a U-turn, and repeated. Obstacle task: Patients walked at a natural speed, stepped over an obstacle, and continued walking normally	Five discrete locomotor states: “standing” (the period between the turning and the gait initiation, or between gait termination and the turning), gait “initiation” (starting 0.5s prior to the first heel-off until the first heel strike), gait “termination” (the last 2 gait cycles), “continuous walking” (all steps in between), and “turning”	Band-pass filter (20–500Hz, 4th-order Butterworth), rectification, and low-pass filter (7 Hz, 4th-order Butterworth)	Synergies were extracted from both legs during small and big steps, with traces time-interpolated over 4 gait phases to obtain average profiles, excluding gait initiation and termination	Envelopes were normalized by their maximum value throughout the session	NMF	>90% of the variance of the original data

#### 3.2.1 Type and number of tasks

Three main tasks were evaluated in the studies: walking at self-selected speed, postural control, resting and reaching movements (Figure 2D). Walking tasks were assessed in five studies; it was possible to separate studies in which the analyzed subjects walked at a self-selected speed on an instrumented treadmill for a specific time (Rodriguez et al., 2013; Roemmich et al., 2014) from studies in which subjects were requested to perform overground walking at a preferred speed along a predefined path (Allen et al., 2017; Thenaisie et al., 2022; Ghislieri et al., 2023). Only Roemmich et al. evaluated both conditions (Roemmich et al., 2014). Moreover, Allen et al. assessed also the reactive balance in addition to the walking task, while Thenaisie et al. added an obstacle along the path to evaluate the subjects’ behavior when they met the obstacle (Allen et al., 2017; Thenaisie et al., 2022). Seven studies considered the postural control task, that consisted in evaluating three body conditions: quiet standing, voluntary sway, and load release (Falaki et al., 2017a; Falaki et al., 2017b; Falaki et al., 2016; Falaki et al., 2023; Freitas et al., 2020; Falaki et al., 2018). In the first condition, subjects stood quietly on the platform for a specific time trying to avoid body movement. The second condition consisted in swaying for 30s mainly about the ankle joints in the anterior-posterior direction maintaining the natural initial position. One of the studies which analyzed this postural control task, added a further condition in which subjects had to reach a posterior target shown on the screen, keep the position, and then perform a discrete body motion forward, paying attention mainly to the speed rather than the accuracy of the movement (Falaki et al., 2017b). Lastly, Mileti et al. assessed postural control asking participants to be subjected to two perturbations with different frequency (Mileti et al., 2020a). Finally, three studies investigated resting and reaching movements in which participants had to start from a resting position, that caused tremor condition, and then reach some target placed in a circle with their hand returning every time at the start position (Bai et al., 2021; Hu Z. et al., 2019; Hu Z.-X. et al., 2019).

#### 3.2.2 Experimental conditions and trials used to extract muscle synergies

The analyzed studies discussed several experimental conditions that can be clustered into four macro-groups. The first cluster considered all studies that investigated walking tasks comparing PD patients after a rehabilitation period, even with respect to healthy controls. Ghislieri et al. (2023) evaluated PD patients during locomotion through instrumented gait analysis at three time points (before surgery, after 3 and 12 months); the control groups performed only the overground walking task once. To extract muscle synergies helpful to compare PD patients at different time points and control groups, the study considered 10 gait cycle time epochs, where each gait cycle included only the straight path discarding the curved trajectories and direction changes at the beginning and at the end of the walkway (Ghislieri et al., 2023). Rodriguez et al. assessed both PD patients and neurologically-healthy older adults while they were walking for 10 min with a preferred speed. To extract muscle synergies EMG data were collected over the last 4 min of the walking (Rodriguez et al., 2013). Moreover, in the study from Thenaisie et al., patients were instructed to stand for about 3 s before initiating a sustained bout of walking at their comfortable speed, placing their feet on marked lines on the floor that distinguished big and small steps. When arriving at the end of the bout, patients were instructed to stop and stand for another 3 s, before doing a U-turn and starting again. To create the matrix for synergies extraction each time-point of the recordings was divided into five discrete locomotor states (“standing,” gait “initiation” and “termination,” “continuous walking,” and “turning”) and for each walking sequence, gait initiation was defined as starting 0.5s prior to the first heel-off until the first heel strike, while gait termination was defined as the last 2 gait cycles. Standing was defined as the period between the end of turning and the beginning of gait initiation, or as the period between gait termination and the beginning of turning (Thenaisie et al., 2022). Finally, Allen et al. analyzed the overground walking at a self-selected speed for every PD patient that had followed a rehabilitation therapy for 3 weeks. Participants were instructed to walk for ∼7.5 m as they would do normally while maintaining their head level and to extract the synergies for this task at least five total gait cycles per walking condition were included. However, this study evaluated also the reactive balance for which they recorded postural responses to ramp-and-hold translations of the support surface during standing while participants stood on an instrumented platform that translated in 12 equally spaced directions in the horizontal plane. The perturbation level was adjusted for each participant such that they could perform the set of perturbations without stepping and this adjustment was repeated both before and after the rehabilitation test. EMG data were analyzed during four different time bins: one before the perturbation and three during the automatic postural response (Allen et al., 2017).

Since Allen et al. assessed also balance control, their study also belongs to the second macro-group of studies that evaluated postural control. Falaki et al. in 2023 analyzed participants who maintained the initial natural standing posture and leaned forward about the ankle joints to shift their center of pressure 3-cm anterior to the initial position asking them to maintain this posture as stable as they could for about 2–3 s while keeping their feet in full contact with the platform, and initiate gait in a self-paced manner by making two steps leading with the right leg. This gait initiation was performed in two conditions, with a quick prior turn of the head (Turn) and without it (no-Turn). Only sway cycles during the middle 25 s of each voluntary sway trial were accepted to avoid the effects of sway initiation and termination (Falaki et al., 2023). Falaki et al. also performed a study where the same quiet standing was analyzed along with voluntary sway task. Instead of starting the gait, participants had to release a load with their arms extended forward and palms facing upward while holding a load. For the voluntary sway task, signals in the interval {3 s; 28 s} were considered for data analysis in order to avoid edge effects and during the fast sway and load release tasks, trials without major errors were accepted (Falaki et al., 2016). In the study of Freitas et al., participants performed the same tasks seen in Falaki et al., but in this study the EMG data obtained from quiet standing trials were used during offline data processing, while the data from voluntary sway trials were later used to identify the jointly activated muscle group (Freitas et al., 2020). Another study performed by Falaki et al. analyzed the same postural control tasks, but the PD patients were subjected to two conditions: DBS-ON and DBS-OFF condition. PD patients performed the tasks before without being subjected to DBS and then after 10 min break, they did the same tasks with the DBS application. Subjects repeated this task for 24 trials with 10-s rest between trials and a 2–3 min break after each set of 12 trials and this data collection was used to extract synergies (Falaki et al., 2018).

The third cluster of studies is composed of all the investigations that compared PD patients that were subjected to their daily medication (ON-drug condition) and PD patients having a medication washout period of at least 12 h (OFF-drug medication). Mileti et al. performed two perturbation tasks at different frequencies around the vertical axis on the transverse plane with a robotic platform. These perturbations were applied first on PD patients in OFF-drug condition and then on patients which were in ON-drug condition. Tasks were repeated three times in a random order (Mileti et al., 2020a). Moreover, Falaki et al. performed two studies where they applied the postural control task testing PD patients in ON-drug and OFF-drug condition (Falaki et al., 2017a; Falaki et al., 2017b). In both studies, the quiet standing task and voluntary sway task were tested and the time interval between two consecutive anterior-posterior COP coordinates was defined as a sway cycle accepting only the data within {3 s; 28 s} time interval for this task. In their second work, they performed more tasks in this order: quiet standing task, voluntary sway task, block randomized load release task, and fast body motion task (Falaki et al., 2017b). Each subject performed 10 full cycles of voluntary sway task and trials without major errors only were accepted. Finally, another study which belongs to this group was performed by Roemmich et al. in which patients had to walk first overground and then on a treadmill on DRUG-OFF condition. Subsequently, 1 h after assuming their daily medication, they walked on a treadmill with the same preferred speed used on drug-off condition and then performed also ten overground gait trials at a self-selected comfortable pace. To collect the data useful to extract muscle synergies all participants first performed ten overground gait trials at a self-selected comfortable pace, then walked on the treadmill for 1 min at 1.0 m/s and at their preferred walking speed for 5 min. Subsequently, patients assumed the medication and after an hour they walked on the treadmill at the same speed they had previously selected while they were in OFF-drug condition. Ten overground gaits performed by patient in ON-drug condition were excluded from the data analysis (Roemmich et al., 2014).

The last group of studies regarded the reaching movement task. Bai et al. analyzed subjects who performed reaching movements to reach a target while they were sitting in front of a frictionless platform with hand placed on an arm support apparatus. To extract tremor-related synergy patterns, the EMGs of distinct tremor cycles between −4 and −2 s prior to the “go” cue were taken to form a data matrix termed “tremor prior”, while the tremor EMGs between 2 and 4 s after compound movement formed another data matrix, termed as “tremor after,” and the EMG data of forward reaching movement between 0 and 2 s after “go” cue formed a data matrix for extraction of compound movement synergy patterns (Bai et al., 2021). Hu et al. designed two studies where PD patients performed targeted reaching movements with no visual feedback of their hand. In the first study (Hu Z. et al., 2019), the bipolar surface electrodes were placed on the dorsal skin of the hand to inhibit tremor during the task, while in the second (Hu Z.-X. et al., 2019) patients performed targeted reaching movements while on and off cutaneous stimulation was delivered to them randomly without informing the subject. In both studies, the length of each tremor cycle was about 550 samples (0.23 s) and for each individual synergy extraction, 2s duration points of EMG signals prior to and during stimulation were selected for the analysis (Hu Z. et al., 2019; Hu Z.-X. et al., 2019).

#### 3.2.3 Data filtering techniques

All the studies followed a specific pipeline for obtaining the envelope of the EMG signal, which consists in removing motion artifacts with band-pass or high-pass filters, rectifying the signal, and computing the envelope with a low-pass filter or a moving average filter. Interestingly, the pipeline was generally different between studies that employed the muscle synergy model or UCM method for the analysis. Most of the studies related to the UCM band-pass filtered the raw EMG signal (20–350 Hz) with a fourth-order, zero-lag Butterworth filter (Falaki et al., 2017a; Falaki et al., 2017b; Falaki et al., 2023; Falaki et al., 2018). Then, the rectified signal was low-pass filtered with a 100 ms window moving average. Additionally, Freitas et al. integrated the normalized EMG signal from each cycle over 50 ms time windows (Freitas et al., 2020), while Falaki et al. (2023) applied an electromechanical delay of 50 ms and averaged over 50 ms windows the EMG and COP data from the voluntary sway task. In some cases, the EMG signal was corrected for baseline values by subtracting the average muscle activation levels measured within the quiet standing tasks (Falaki et al., 2017a; Falaki et al., 2017b; Falaki et al., 2016). Studies in which muscle synergy model was applied employed different pipelines. Three studies used a notch-filter at 50 and 120 Hz with their harmonics to remove the noise of the power line and magnetic field of the environment, then a band-pass filter (20–390 Hz) and, after the rectification, a low-pass filter with a cut-off frequency of 20 Hz. This type of filtering was applied typically in studies that analyzed reaching movement where also the tremor activity was assessed (Bai et al., 2021; Hu Z. et al., 2019; Hu Z.-X. et al., 2019). In particular, in the two studies by Hu et al., the tremor group was aligned at the peak value of the first muscle EMG and EMG envelopes were aligned, in the first case, at the peak of hand velocity, with a time duration of 0.5 s prior to peak velocity and a time duration of 1.3 s after peak velocity (Hu Z. et al., 2019); and in the second case, at movement initiation, with a time duration of 0.25s prior to movement initiation and a time duration of 0.95 s after movement initiation (Hu Z.-X. et al., 2019). Four studies used a high-pass filter with a cut-off frequency of 35 Hz, but they used different methods for obtaining the envelope: Rodriguez et al. and Roemmich et al., who evaluated the walking task on a treadmill, demeaned, rectified the filtered EMG and applied a zero-lag 4^th^-order low-pass Butterworth filter at 7 Hz (Rodriguez et al., 2013; Roemmich et al., 2014); Allen et al. demeaned, rectified, and low-pass filtered the signal at 40 Hz (Allen et al., 2017); Mileti et al. applied to the EMG filtered a notch filter at 50 Hz to remove power-line artifacts, rectified the signal, and then the envelope obtained was low pass filtered with a cut-off frequency of 5 Hz. Moreover, in this study, all signals were resampled to have 100 samples in total (Mileti et al., 2020a). Thenaisie et al. treated the raw signal with a band-pass filter (20–500Hz, zero-lag 4th-order Butterworth filter), rectified the signal and applied a zero-lag 4th-order low-pass Butterworth filter at 7 Hz (Thenaisie et al., 2022). Finally, Ghislieri et al. filtered the raw signal with a 2nd zero-lag IIR band-pass Butterworth filter between 10 and 500 Hz. Then, the EMG signal was high-pass filtered (8th order zero-lag IIR Butterworth digital filter with a cut-off frequency of 35 Hz), rectified, and low-pass filtered (4th order zero-lag IIR Butterworth digital filter with a cut-off frequency of 12 Hz) (Ghislieri et al., 2023).

Although each study used different techniques or cut-off frequency, the steps of the pipeline follow the same aims: the first step is needed to remove motion artifacts and the noise of the power line and magnetic field of the environment; then, the rectification allows to make the signal fully positive; the last step is needed to smooth the signal in order to have the envelope. The different cut-off frequencies may be based on the type and the amount of noise of the data in the specific study.

#### 3.2.4 Data stacking technique

**FIGURE 3:**
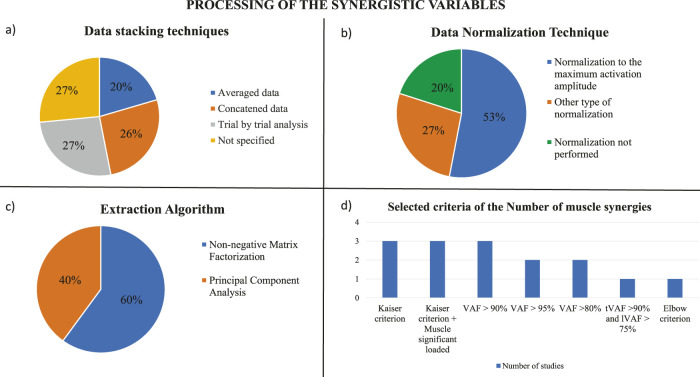
Processing of the synergistic variables. **(A)** Data stacking techniques used in the screened studies. **(B)** Techniques of data normalization which were applied by the studies analyzed. **(C)** Extraction algorithms used in the screened studies. **(D)** Selected criteria applied by the studies analyzed to choose the proper number of muscle synergies.

The stacking technique describes how EMG data are organized within the matrix before synergy extraction. EMG data can be stacked in three different ways: they can be averaged, concatenated, or considered with a trial-by-trial analysis (Figure 3A). The screening revealed that in four studies the EMG data were concatenated to create the matrix from which synergies were extracted. In particular, Ghislieri et al. concatenated 10 gait cycles into a time epoch (Ghislieri et al., 2023); Bai et al. concatenated EMGs of distinct tremor cycles and the EMG data of forward reaching movements in distinct matrices (Bai et al., 2021); Freitas et al. concatenated data to create a matrix with each column corresponding to a muscle and the rows corresponding to the samples across the sway cycles analyzed (Freitas et al., 2020); and, finally, Allen et al. concatenated the EMG data only for walking task (Allen et al., 2017). Another option is to average the data as in Mileti et al. and Hu et al. where the EMG matrix of all the trials for synergy analysis was built by averaging activation signals between three repetitions and averaging the tremor group of each trial, respectively (Mileti et al., 2020a; Hu Z. et al., 2019; Hu Z.-X. et al., 2019). Also, Allen et al. considered balance assessment by assembling the mean muscle activity values for each muscle during each bin during each trial to form an *m* × *t* data matrix (Allen et al., 2017). Finally, in four studies a trial-by-trial analysis was performed to create the matrix for the synergy extraction (Falaki et al., 2017a; Rodriguez et al., 2013; Roemmich et al., 2014; Thenaisie et al., 2022). In particular, Rodriguez et al. and Roemmich et al. performed a trial-by-trial analysis evaluating each gait cycle separately with the assumption that muscle weightings were fixed for that cycle while activation profiles were allowed to vary across gait cycles (Rodriguez et al., 2013; Roemmich et al., 2014). Thenaisie et al. considered all muscle envelopes of the left and right legs together, using recordings from both the small and big stepping tasks. Then to obtain average synergy activation profiles for each task, they linearly time interpolated synergy traces over the 4 phases of each gait cycle (stance, swing, and double stance for each leg, as defined by gait events) and gait initiation and termination steps were excluded from this average (Thenaisie et al., 2022).

The different stacking techniques may be related to the type of data and the aim of the analysis. In particular, the concatenation allows to preserve the variability of the data during the task which is an important factor for muscle synergy analysis; however, the data may be noisy. The averaging technique reduces the noise of the data, losing the natural variability of movement repetitions. Finally, the trial-by-trial data analysis allows to assess the intra-subject repeatability and to analyze different tasks separately; however, the data may capture single trial noise.

#### 3.2.5 Data normalization

The aim of the normalization is to change to a common scale the values of the numerical columns in the data matrix. Normalization is needed when the data distribution is unknown and it is very useful to compare several data which belong to different scales; in fact, normalization allows intra-subject comparisons in different sessions and inter-subject comparisons. It is possible to apply different techniques to normalize EMG data. In this review, eight studies normalized EMG data to the maximum activation amplitude observed for each muscle across all trials (Mileti et al., 2020a; Allen et al., 2017; Falaki et al., 2017b; Falaki et al., 2016; Rodriguez et al., 2013; Roemmich et al., 2014; Thenaisie et al., 2022; Ghislieri et al., 2023) (Figure 3B). Other four studies normalized the EMG envelope with the following relation

$$EMG_{norm}=\frac{EMG-EMG_{QS}}{{EMG}_{\max}}$$

where EMG_QS_ denoted the averaged EMG activity of each muscle during the middle 10 s of the quiet standing task and EMG_max_ was the maximal activation level of the corresponding muscle across voluntary sway and gait initiation tasks (Falaki et al., 2017a; Falaki et al., 2023; Freitas et al., 2020; Falaki et al., 2018). However, in some studies, no normalization of EMG data was performed or declared (Bai et al., 2021; Hu Z. et al., 2019; Hu Z.-X. et al., 2019).

#### 3.2.6 Extraction algorithm and selection of the number of synergies

In the screened studies, two methods were employed for extracting synergies: the PCA and the NMF, that is the most widely used method in the literature for synergy extraction (Mileti et al., 2020b). In particular, six articles used PCA with Varimax rotation and factor extraction and nine studies applied the NMF (Figure 3C). PCA was principally used in the studies where postural control and oscillations of the center of pressure of the subjects were assessed with the UCM method, while the NMF was used when walking tasks and reaching movements were evaluated with the muscle synergy method. To select the number of muscle synergies to consider during the different assessments, several criteria were defined by the studies (Figure 3D). Studies that used the PCA method with Varimax rotation and factor analysis to extract muscle synergies, applied the Kaiser criterion to select as M-modes the first four PCs with the greatest eigenvalues and confirmed this choice by visual inspection of the scree plot (Falaki et al., 2017b; Falaki et al., 2016; Falaki et al., 2018). In addition, some of these types of studies added another criterion to define the M-modes: at least one muscle had to be significantly loaded (load higher than ±0.5) in at least one of the four PCs, in this way each M-mode had to contain at least one significantly loaded muscle (Falaki et al., 2017a; Falaki et al., 2023; Freitas et al., 2020). For studies which used NMF, the criterion to select the proper number of muscle synergies was based on the analysis of the Variance Account For (VAF) curve, that defined how well the number of selected synergies reconstructed the EMG input. In each study, a threshold had to be reached to find the correct compromise between the computational effort and the accuracy of reconstruction. Three of the studies selected a threshold for VAF greater than 90% (Allen et al., 2017; Bai et al., 2021; Thenaisie et al., 2022). Two studies that analyzed the reaching movements selected the number of synergies when the VAF of more than half of patients exceeded 80% (Hu Z. et al., 2019; Hu Z.-X. et al., 2019), while the other two studies assumed that the reconstruction was acceptably accurate if the total %VAF across all reconstructions collectively reached 95% (Rodriguez et al., 2013; Roemmich et al., 2014). Moreover, Mileti et al. identified the minimum number of synergies for each task and all groups by following these criteria: the total VAF greater than 90%, and the local VAF greater than 75%, which was the correlation between acquired signal with the reconstructed one for each muscle (Mileti et al., 2020a). Finally, Ghislieri et al. used the so-called elbow criterion that was implemented to avoid setting arbitrary cut-off thresholds on the model reconstruction quality (Ghislieri et al., 2023).

### 3.3 Synergistic results

Synergistic results are shown in Table 4.

**TABLE 4:** Results obtained from the analysis of synergies extraction.

First author	Year	Number of extracted synergies	Spatial synergies variation (Δ*W)*	Temporal coefficients variation (Δ*C)*
Ghislieri et al.	2023	4 synergies in patients at each T and 5 synergies in controls The number of muscle synergies was lower in PD patients at T0, at T1, and at T2 than control subjects. Muscle synergies related to body stabilization and dynamic postural control appear to be the most affected by muscle synergy merging observed in PD patients	Increased muscle coactivation in PD patients compared with healthy subjects. The neuromuscular robustness (CrossVAF) of PD patients was reduced with respect to controls, and increases after DBS, becoming not different from that of controls at T2, suggesting an improvement of smoothness and a decrease of variability of neural commands	Reduced independence of neural control signals in PD patients compared to healthy subjects
Bai et al.	2021	3 muscle synergies were extracted	One pair of synergy vectors only changed in transition, while the other two pairs were almost identical with a similarity index >0.97. The third in compound movement showed an increase in activation of Tri muscle in almost all trials, needed to accelerate and decelerate the arm. The two unchanged synergies indicated that tremor activity co-exists with movement activity, suggesting that pathological tremor modules may share the same neural substrate that generates voluntary movement	The first temporal coefficient showed a decrease in activation level, which may indicate an inhibition of the tremor generator module by the movement module. The second one elevated slightly in amplitude, and the third one presented little oscillation component. After compound movement activity disappeared, and tremor amplitude was reduced slightly
Mileti et al.	2020	The median was 4 for all tasks and PD patients’ groups (both ON and OFF conditions) while was 5 for all tasks for the control groups. Low frequency perturbation: 3 to 6 for PD in the OFF condition and 4 to 7 for PD in the ON condition and case controls. High frequency perturbation: 2 to 6 in PD patients in both ON and OFF conditions and 2 to 5 in the control group PD patients exhibited higher values of tVAF across all number of synergies compared to healthy subjects. Patients with higher cognitive deficits recruited a lower number of muscle synergies	—	—
Hu et al.	2019	3 muscle synergies were extracted	Cutaneous stimulation did not notably alter the synergy vector patterns both for tremor and voluntary movement. All patients had a vector synergies similarity index >0.900 both for tremor and voluntary movements	Cutaneous stimulation did not alter the time profiles of tremor synergy but reduced the amplitudes for the two main components. The amplitudes in time profiles of voluntary movements were reduced. The time profile had a similarity index >0.9 for tremor, while the similarity indices had a range from 0.5 to 0.7 for the voluntary movement
Hu et al.	2019	3 synergies for tremor activities. 3 synergies for voluntary movements and the number of synergies did not change after therapies	For the voluntary movement, one vector only was altered by the stimulation in both PD and control subjects. In tremor synergies, there was no difference in vector patterns and the similarity indices were greater than 0.900 in all the PD patients, both before and during the cutaneous stimulation. For voluntary movement, the similarity indices were greater than 0.900 in all the control and PD subjects	In controls, time profiles with and without stimulation were similar, while in PD patients, stimulation reduced peak amplitude. Stimulation lowered the PSD of muscle synergy time profiles in both groups during tremor and voluntary movement. For tremor, eight of ten PD patients had similarity indices >0.900, with one at 0.478 and an average of 0.865 during stimulation. For voluntary movement, similarity indices were <0.900 in nine of ten PD patients and ≥0.900 in seven of eight controls
Allen et al.	2017	Extracted synergies: 4–5.3 patients had the same number of synergies, while 3 decreased	Motor modules became more consistent and distinct after AT, and most participants decreased motor module variability and increased motor module distinctness in both walking and reactive balance. Most participants increased motor module coactivity after short-term rehabilitation. Post rehabilitation there was an increased percentage of shared synergies between walking and reactive balance	—
Rodriguez et al.	2013	PD patients required fewer modules than controls to achieve 95% VAF. Of 30 PD legs, 3.3% required three modules, 83.3% required four modules, and 13.3% required five modules; of 28 control legs, 7.1% required three modules, 50% required four modules, 35.7% required five modules, and 7.1% required six modules. On average, 4–5 modules were considered both for PD and for controls	The muscle weightings in each module were comparable between groups; the contributions of each muscle to PD modules were very similar to control modules when the number of modules was constant. The NMF analysis was able to reconstruct GAS, SM, and BF signals more accurately in PD compared to controls when the number of modules was constant	When 4 modules were extracted, the first peak of the third module occurred later in PD, with a lower and delayed second peak. The fourth module peak was also lower in PD. When 5 modules were extracted, the second peak of the second module, the peak of the fourth module, and the first peak of the fifth module occurred later in PD. The first peak of the third module was higher, while the peaks of the fourth and fifth modules were lower. Additionally, the first peak of the third module occurred later, and the second peak was lower in PD.
Roemmich et al.	2014	No significant differences in total %VAF were found between OFF meds Pref and ON meds Pref. All participants reached 95% VAF with four or five modules. No significant differences in %VAF between the medicated states. No significant differences when comparing %VAF with four modules between OFF meds Pref and OFF meds Fast. A significant difference was found when comparing OFF meds Pref to OFF meds OG in both total %VAF.	No differences in the structure of the modules, in the sense of the individual contributions from each muscle to the muscle weighting vectors, in the four-module configuration between OFF meds Pref and ON meds Pref.	There was only a significant increase in the magnitude of the first peak of the activation profile of the third module during ON meds Pref compared to OFF meds Pref, while there were not any other differences between ON meds Pref and OFF meds Pref in the timing or amplitude of any other module’s activation profiles when assuming four modules
Thenaisie et al.	2022	4 muscle synergies were extracted	All four synergies exhibited an increase in force. Lengthening the step during transitions from a normal to a long step involved a significant increase in the amplitude of muscle synergies associated with ipsilateral propulsion and contralateral weight acceptance	The timing of these synergies did not significantly change in synergies 1,2 and 4 if considered walking with different lengths of steps, while the third synergies activated later in the long step compared to the short one

#### 3.3.1 Number of extracted synergies

To assess the walking tasks in order to compare healthy controls and PD patients or to evaluate if PD patients improved their conditions after the rehabilitation treatment, the number of extracted synergies was mostly 4-5 synergies. PD patients might require fewer modules to reach the VAF threshold to obtain the optimal reconstruction of the original EMG signal and it was also observed that cognitive deficit might affect the number of synergies. In fact, the higher the cognitive deficit was, the lower the number of recruited synergies was (Mileti et al., 2020a). According to Rodriguez et al., to achieve 95% VAF, considering 30 PD legs, 3.3% required three modules, 83.3% required four modules, and 13.3% required five modules; while considering 28 healthy controls legs, 7.1% required three modules, 50% required four modules, 35.7% required five modules, and 7.1% required six modules; so, people with PD generally required fewer modules compared to healthy controls (Rodriguez et al., 2013). Moreover, it was observed that PD patients both in OFF and ON conditions exhibited higher values of tVAF compared to healthy subjects. In addition, a higher value of coefficient of variability was reached by the healthy adults while a lower value of variability was reported for patients with Parkinson’s disease (Mileti et al., 2020a). On the contrary, when comparing PD patients in ON-drug condition and PD patients in OFF-drug condition, there was no significant difference in the total %VAF for any number of modules or a difference in the proportion of legs requiring four or five modules to reach 95% VAF and there were also no significant differences between medicated states in the %VAF for individual muscle EMG signals reconstruction (Roemmich et al., 2014). Also, after a period of rehabilitation there were no differences between the number of extracted synergies, and only few subjects showed a decrease in the number of synergies (Allen et al., 2017). In another study, it was noted that DBS treatment did not affect the number of muscle synergies extracted; in fact, the number of synergies remained lower in PD patients subjected to DBS compared to healthy subjects. In addition, the same studies showed that muscle synergies related to body stabilization and dynamic postural control appeared to be the most affected by muscle synergy merging observed in PD patients (Ghislieri et al., 2023). In reaching movements, the number of extracted synergies was usually 3 as shown by three studies which evaluated the influence of tremor on voluntary movement (Bai et al., 2021; Hu Z. et al., 2019; Hu Z.-X. et al., 2019). In particular, Hu et al. extracted 3 synergies to characterize tremor activities and 3 synergies to describe voluntary movements, and they showed that the number of synergies did not change after therapies (Hu Z.-X. et al., 2019).

Although the number of EMG channels included in the analysis was different among studies, most of them extracted a similar number of synergies (3 for reaching movements and 4-5 for walking tasks), independently of the number of EMG channels. Moreover, PD patients showed fewer synergies with respect to healthy control, suggesting a reduced complexity of motor control. Indeed, the rigidity and the reduced coordination led to increase muscle co-contraction and the difficulty of motor control of recruiting specific muscles. The ability of the CNS to independently control muscle synergies related to different biomechanical functions is strongly reduced, revealing a lack of modular independence and therefore a reduced motor control complexity (Ghislieri et al., 2023).

#### 3.3.2 Spatial synergies variation (ΔW)

In reaching movements, studies investigated synergies in tremor and voluntary movement, extracting three synergies. One study showed that during the transition from resting tremor to subsequent compound movement the third synergy only was the most affected by movement, showing an increase in activation of the triceps muscles for the acceleration and deceleration of the movement and a significant drop in the similarity of the third synergy (Bai et al., 2021). The two unchanged synergies indicated that tremor activity co-exists with movement activity, suggesting that pathological tremor modules may share the same neural substrate that generates voluntary movement. Two studies investigated the effects of hand cutaneous stimulation, finding that for the voluntary movement, the 1st and 2nd synergies were similar to each other both with and without hand cutaneous stimulation, while the 3rd vector pattern was altered subtly by the stimulation in both PD and control subjects (Hu Z. et al., 2019). In these studies, the authors also demonstrated that for the tremor activities, the similarity indices of the synergies with and without stimulation were greater than 0.90 in all the PD patients, showing that the cutaneous stimulation did not alter the synergy vector patterns notably both for tremor and for voluntary movements (Bai et al., 2021; Hu Z. et al., 2019; Hu Z.-X. et al., 2019).

In walking tasks, comparing healthy participants and PD patients subjected to DBS during walking tasks, a study showed an increased muscle coactivation in PD patients compared to healthy subjects, related to the rigidity and the inability to recruit specific muscles as already shown also in the lower number of synergies. In addition, the neuromuscular robustness of PD patients, defined by the CrossVAF, was reduced with respect to controls; it increased after DBS, becoming not different from that of controls 12 months after surgery. This result documented an improvement in smoothness and a decrease in variability of neural commands used during the motor tasks (Ghislieri et al., 2023). Observing how muscle synergies changed after a period of rehabilitation, Allen et al. showed that motor modules became more consistent and distinct after an Adaptive Tango course, used as rehabilitation therapy. Participants decreased motor modules’ variability and increased motor modules’ distinctness in both walking and reactive balance. Moreover, it was observed that post rehabilitation led to an increased percentage of shared synergies between walking and reactive balance, suggesting an increased modules coactivity for both tasks (Allen et al., 2017). When comparing the weights within any of the modules’ muscle weight vectors between PD patients and healthy controls or between PD patients in ON-drug condition and in OFF-drug condition, the investigators did not notice any difference between the two groups. Hence, the muscle weights in each module were comparable between groups and this means that the contributions of each muscle to PD modules were very similar to the contributions of each muscle to healthy controls modules when the number of modules was constant (Rodriguez et al., 2013; Roemmich et al., 2014).

Finally, Thenaisie et al., considering patients who walked with different length strides, discovered that lengthening the step during transitions from a normal to a long step involved a significant increase in the amplitude of the weights of muscle synergies associated with ipsilateral propulsion and contralateral weight acceptance (Thenaisie et al., 2022).

#### 3.3.3 Activation coefficient variation (ΔC)

In reaching movement tasks, several studies noted differences in amplitude and activation level of the time profile of muscle activation. In particular, Bai et al. investigated how the synergy patterns from resting tremor to subsequent compound movement changed. They observed that the time profile of the first synergy showed a decrease in activation level, indicating an inhibition to the tremor generator module by the movement module, the time profile of the second synergy elevated slightly in amplitude, and the third time profile presented little oscillation components (Bai et al., 2021). Other observations were made to evaluate the influence of cutaneous stimulation on the inhibition of the tremor component during voluntary movements, and it was discovered that cutaneous stimulation did not alter the time profiles of tremor synergy, but reduced the amplitudes for the two main components. The influence of cutaneous stimulation on voluntary movements changed when considering PD patients or case controls. In fact, in control subjects, the time profiles of voluntary movements were similar to each other with or without stimulation, while in PD patients, cutaneous stimulation reduced the amplitude of the peak of time profile. Moreover, it was observed that cutaneous stimulation reduced the power spectral densities (PSD) of time profiles of muscle synergies in both control subjects and PD subjects, and both in tremor and voluntary movements (Hu Z. et al., 2019; Hu Z.-X. et al., 2019). The reduction of PSD of time profiles may be correlated to the prolongation of movement time in reaching movement and the inhibition of tremor.

Comparing healthy subjects and PD patients while walking on a treadmill, Rodriguez et al. observed that the first peak in the activation profile of the module controlling the hip in early stance occurred significantly later in the gait cycle of PD patients with respect to healthy subjects, and the magnitude of the second peak of the same module was lower and temporally delayed. Further, the magnitude of the peak of the module responsible for controlling terminal swing was lower in PD patients compared to healthy controls. When extracting five synergies, they discovered that the temporal peaks of the modules controlling swing phase, terminal swing, and early stance occurred significantly later in the gait cycle in PD patients. Further, the magnitude of the first peak of the module controlling the hip was significantly higher in PD subjects, while the peaks of the modules linked to the terminal swing and to the early stance were significantly lower (Rodriguez et al., 2013). Changes in amplitude and shifts in time of the temporal coefficients provide another evidence of the reduced complexity of the neuromuscular control of PD patients. Moreover, Ghislieri et al. noticed that the activation coefficients of synergy in PD patients showed a reduced sparseness with respect to healthy controls, indicating reduced independence of neural signals (Ghislieri et al., 2023). Another study also noted that comparing PD patients in ON-drug condition and OFF-drug condition during walking tasks, there was only a significant increase in the magnitude of the first peak of the activation profile of the module that controls the knee and the hip at the beginning of the gait cycle (Roemmich et al., 2014). Patients showed that the timing of the modules was unaffected by dopaminergic medication, suggesting that the simplification of neuromuscular complexity during gait in PD cannot be treated by dopaminergic therapy alone. When considering different stride lengths during walking, a study observed that only the third synergy activated later in long strides compared to the short ones, while the other synergies did not show any particular changes in the time profile (Thenaisie et al., 2022).

### 3.4 Results in the framework of UCM hypothesis

The results in the framework of the UCM are reported in Table 5.

**TABLE 5:** Results in the framework of UCM.

First author	Year	M-modes	VUCM and VORT	Synergy indices ΔVz	Anticipatory synergy adjustments
Falaki et al.	2023	For each participant, 4 M-modes were identified. The M-modes in the AP direction showed similar amounts of VAF in the original EMG space for the PD, age-matched control, and young control groups. Smaller VAF amounts were seen for the ML direction. Larger total amount of variance in the M-mode space during the Turn than the no-Turn condition	Steady-state condition: VUCM was significantly larger than VORT, particularly pronounced in the young group. VORT was lower during the no-Turn than Turn condition. Across groups, the indices of variance were larger in the Turn than the no-Turn condition	Steady-state: the synergy index was higher in age-matched and young controls than PD. ΔV was higher during the no-Turn than Turn condition, as well as for the ML direction compared to the AP direction. Smaller ΔV in the PD group during the Turn than the no-Turn condition for both AP and ML directions. In the age-matched controls, ΔV was smaller during the Turn condition only in the ML direction, while no significant difference in young controls	ASA smaller in the age-matched controls. ASA magnitudes were bigger in the no-Turn than Turn condition, and in the ML than the AP direction. Young participants showed a larger ASA than age-matched controls and patients. ASAs were also greater in the age-matched controls than the PD group. Among PD patients, ASAs became smaller and even negative during the Turn conditions for both AP and ML directions. No differences in ASA index in the young control group between the no-Turn and Turn conditions
Freitas et al.	2020	4 M-modes were identified from the VS task. The total VAF of these modes was greater for the controls than the PD group. There was only a small, non-significant increase in the VAF after taking the first drug dose compared to the “off-drug” condition	Levodopa-naïve patients had a significantly lower VUCM than controls, suggesting insufficient inter-trial variance of muscle activation pattern. VORT was similar between the groups. VORT was smaller in “on-drug” condition, while VUCM did not change. The first dose of drug produced a drop in VORT and no change in VUCM	The index of synergy was negative for the PD group, whereas it was positive for the controls. The drug led to a higher ΔVZ index compared to the “off-drug” state	The time of APA initiation was delayed significantly in the PD group compared to the control group. After the first dose of medication, the APA initiation time was about 20 ms earlier compared to the “off-drug” state, but this difference was non-significant. Significantly shorter APAs in levodopa-naïve PD patients were observed
Falaki et al.	2018	4 M-modes were identified in each condition. Muscle synergies account for similar amounts of variance in both conditions: DBS-OFF and DBS-ON.	There was no statistically significant difference during SS between the two DBS conditions in either VUCM or VORT.	Synergy indices decreased significantly in PD subjects without DBS treatment and this was demonstrated especially in the postural task. Patients showed significantly smaller indices of multi-muscle synergies stabilizing COP_AP during SS in both DBS-off and DBS-on conditions than controls	There was an increase in indices of ASAs in the DBS-on state compared to the DBS-off state. Significant differences in the ASA parameters between patients and controls. Controls demonstrated significantly larger values of ΔV_ASA compared to both DBS-off and DBS-on state. The ASAs started earlier in controls than DBS-off only, while the difference between controls and DBS-on was non-significant
Falaki et al.	2017	4 M-modes in all subjects. The composition of M1-mode and M2-mode was similar in all subjects and did not differ in PD patients. M3 and M4 compositions were more variable across subjects. In Experiment 1, the four M-modes accounted for less variance in the PD group than controls. In Experiment 2, the M-modes accounted for less variance in the off-drug than the on-drug condition	Experiment 1: both VUCM and VORT in the PD group were smaller than controls. VORT showed a significant phase effect, instead the VUCM was not affected across the Phase. Experiment 2: dopaminergic medications led to an increase in the amount of VUCM without a change in VORT. On average, VUCM in Phase 2 was smaller than in Phase 3	—	—
Falaki et al.	2017	4 M-modes were identified in each condition. The total variance of M-modes increased in ON-drug condition than OFF-drug condition. The on-drug condition showed an increase in variability at the level of M-modes	Steady-state condition: VUCM was higher in the on-drug than the off-drug condition, whereas there were no major differences between the VORT magnitude. For load release (T0), there was a small drop in VUCM and a small increase in VORT in the on-drug condition, without a difference in the off-drug condition. Larger change in the VUCM in on-drug	Nine out of 10 subjects showed a >40% increase in the magnitude of the synergy index from the off-drug to the on-drug condition. ΔVZ during steady state was significantly lower in the off-drug than the on-drug condition	ASAs were significantly smaller in the off-drug condition compared to on-drug. On the contrary, the timing of APAs did not show a significant medication effect on their index
Falaki et al.	2015	4 M-modes were identified in all subjects. PD patients showed a significantly smaller amount of variance. PD subjects showed significantly reduced indices of M-mode synergies stabilizing COP_AP.	—	The synergy index (ΔVZ) was significantly lower in PD subjects compared to controls. There was a larger drop in the synergy index in controls from steady state to the moment of load release, while this drop in the synergies index was absent in the PD group	It was observed significantly reduced ASAs in PD subjects

#### 3.4.1 M-modes

In all the studies which analyzed muscle synergies based on the UCM hypothesis, four M-modes were identified for all subjects. In all these studies, the total VAF of four modes was greater for the controls compared to the PD groups, and this relation was also observed when PD patients were compared in ON-drug and OFF-drug condition, showing that the total variance of M-modes increased in ON-drug condition than OFF-drug condition (Falaki et al., 2017a; Falaki et al., 2017b; Freitas et al., 2020). Another finding was that PD subjects showed significantly reduced indices of M-mode synergies stabilizing the COP anterior-posterior shift, but the variability at the level of M-modes increased when assuming dopaminergic medications (Falaki et al., 2017b; Falaki et al., 2016). Evaluating how the behavior of M-modes changes during the sway of body along anterior-posterior (AP) direction and along medio-lateral (ML) direction, it was noted that the four M-modes in the AP direction and the ML direction showed similar amounts of VAF in the original EMG space for PD patients, aged-matched healthy controls and young healthy controls, but a smaller VAF amounts for all three groups were found for the ML direction compared to the AP direction. Further, there was a larger total amount of variance in the M-mode space during the Turn condition as compared to the no-Turn condition (Ghislieri et al., 2023). Finally, a study verified that DBS surgery did not affect the total VAF, such that muscle synergies account for similar amounts of variance in both conditions: DBS-OFF and DBS-ON (Falaki et al., 2018).

#### 3.4.2 VUCM and VORT

When assessing the postural control based on the shift of the center of pressure, in the framework of the UCM hypothesis, researchers evaluated the variance along the UCM (VUCM), which led to an unchanged center of pressure, and the variance orthogonal to the UCM (VORT), which led to a change of center of pressure. In the studies where PD patients in ON and OFF drug conditions were compared while they were in steady state, it was seen that VUCM was significantly higher in the on-drug condition compared to the off-drug condition, whereas there were no major differences between the VORT magnitude (Falaki et al., 2017a; Falaki et al., 2017b). When comparing PD patients in the off-drug condition and healthy case controls, it was observed that VUCM was smaller in the PD group compared to the control group, while VORT was similar between the groups. When PD patients assumed their first dose of medication, VORT decreased, while VUCM remained unchanged (Freitas et al., 2020). In addition, during this comparison between PD patients and healthy controls, it was explored the phase effect, so that the dependence of VORT and VUCM on the phase of swaying that it divided into four levels in order to represent four windows with the same length in which the sway cycle was divided. It was discovered that VORT showed a significant phase effect, instead the VUCM was not affected across the phase, except when PD patients assumed dopaminergic medication because it was observed a VUCM decrease in phase 2 (Falaki et al., 2017a). During steady state condition, it was noted that VUCM was significantly larger than VORT both in PD patients and healthy case controls and this difference was particularly pronounced when the comparison was done between PD patients and a young group of healthy controls. In all the studies, it was shown that VUCM was lower in PD patients with respect to controls and it increased when the dopaminergic medication was administered, indicating that it influenced motor control. In particular, a higher VUCM was related to higher stability. In addition, a lower amount of VORT was observed during the no-Turn than Turn condition and across groups, the indices of variance were larger in the Turn condition compared to the no-Turn condition (Falaki et al., 2023). Moreover, it was discovered that by the time a load was released, there was a small drop in VUCM and a small increase in VORT in the on-drug condition, without consistent difference in either index in the off-drug condition (Falaki et al., 2017b). Finally, it was noted that the DBS treatment did not affect either VUCM or VORT indices in a statistically significant way (Falaki et al., 2018).

#### 3.4.3 Synergy index ΔVz

Muscle synergies stabilizing the center of pressure shifts along AP direction (COP_AP) were quantified from the inter-trial variance in the muscle mode space to generate variance values that did not affect COP_AP (VUCM) and variance that did (VORT). If VUCM was greater than VORT, synergies could stabilize COP_AP, and in addition an index of synergies ΔVz was calculated as:

$$∆Vz=\frac{\left(VUCM-VORT\right)}{Vtot}$$

where Vtot is total variance.

During steady state condition, it was found that ∆Vz was higher in healthy case controls compared to PD patients, also when PD patients were subjected to DBS treatment (Falaki et al., 2016; Falaki et al., 2023; Falaki et al., 2018).

A study which investigated the COP stabilizing in the AP direction and in the ML direction while PD patients turning or not their heads, noted that in all groups ∆Vz was higher during the no-Turn than Turn condition, as well as for the ML direction compared to the AP direction. Further, ∆Vz was smaller in the PD group during the Turn condition compared to the no-Turn condition for both AP and ML directions and in the age-matched controls, ∆Vz was smaller during the Turn condition only in the ML direction, while there was no significant difference in the young healthy controls (Falaki et al., 2023). It resulted that ∆Vz was negative for PD patients, whereas it was positive for healthy controls (Freitas et al., 2020). Moreover, some studies compared PD patients that assumed their medication regularly and PD patients in OFF-drug condition and they observed that during steady state condition ∆Vz was significantly lower in the off-drug condition compared to the on-drug condition. In fact, drug led to higher ∆Vz index compared to the off-drug state as it was seen in a study where nine patients out of ten showed a 40% increase in the magnitude of the synergy index from the off-drug condition to the on-drug condition (Falaki et al., 2017b; Freitas et al., 2020). Finally, when patients performed a load release task, by the time they passed from steady state to the moment of load release a larger drop in the synergy index in control subjects was observed, while in PD patients this drop was absent (Falaki et al., 2016).

#### 3.4.4 Anticipatory synergies adjustments

In a variety of multidigit and multi-joint tasks associated with self-initiated perturbations, anticipatory synergy adjustments (ASAs) were investigated. The aim of ASAs seems to be the attenuation of synergies that otherwise interfered with the planned quick change of the performance variable. So, ASAs were observed when subjects performed actions that involved a quick change of a redundant set of variables (Klous et al., 2011). The investigation of PD patients and healthy subjects postural control noted that ASAs index varied in the two groups. Comparing PD patients to aged-matched healthy subjects and to young healthy controls, a study observed that young controls showed larger ASAs than aged-matched controls and patients, but at the same time, the aged-matched controls had ASAs index greater than PD patients. Furthermore, considering the COP sway along AP and ML direction with Turn or no-Turn of the head, it was observed that ASAs magnitudes were on average bigger in the no-Turn than Turn condition, and in the ML direction compared to AP direction. Further, ASAs for PD patients became smaller and even negative during the Turn conditions for both AP and ML directions (Falaki et al., 2023). There was an increase in indices of ASAs in the DBS-on state compared to the DBS-off state and in ON-drug condition compared to OFF-drug condition. The DBS state influenced the ASAs initiations, whereas the medication did not show any effect on it. In fact, the ASAs started earlier in controls compared to DBS-off only, while when PD patients were subjected to DBS there was no difference in ASAs timing with controls, although the controls demonstrated significantly larger values of ASAs compared to both DBS-off and DBS-on state (Falaki et al., 2017b; Falaki et al., 2018). Higher ASAs seemed to be related to higher agility and to a better feed-forward control of multi-muscle stability and, therefore, both DBS and medications resulted in improved agility and control.

Additionally, also anticipatory postural adjustments (APAs) timing was evaluated. APAs were detected when a standing person has to perform a fast movement and a change in the activation of postural muscles is observed before the movement starts. So APAs’ aim has been assumed to produce joint torques and forces that minimize perturbation of the vertical posture that would otherwise be associated with the movement (Klous et al., 2011). Freitas et al. observed that the time of APAs initiation was delayed significantly in the PD group compared to the control group. After the first dose of medication, the APAs initiation time was about 20 milliseconds earlier compared to the off-drug state, but this difference was non-significant, but significantly shorter APAs in levodopa-naïve PD patients were observed (Freitas et al., 2020).

## 4 Discussion

### 4.1 Methodological considerations

Since this review aims at covering methodological issues, we provide a list of relevant considerations and suggestions regarding the use of muscle synergies in PD.

#### 4.1.1 Two different muscle synergy concepts

We found that the use of muscle synergies divides into two different methodological approaches. The first approach considers muscle synergies as a dictionary of co-activating patterns coded at neural level that can reconstruct the variety of muscle patterns underlying motor control (Falaki et al., 2017a; D’Avella et al., 2003). The second approach is based on the principle of motor abundance and considers synergies as a neural organization of abundant sets of elements that have to provide stability of silent performance variables. In our screening, we noted that the first approach has been used to evaluate the walking tasks and reaching movements (e.g., Bai et al., 2021; Rodriguez et al., 2013) because during these tasks the CNS has to control and coordinate the building blocks underlying movements. To extract muscle synergies this approach uses the NMF algorithm based on the time-invariant model which allows to express the EMG activity as a linear combination of time-invariant synergies with a fixed weight among muscle (scalar values activated at the same time) multiplied by a set of time-varying activation coefficients (Ebied et al., 2018). Instead, the second approach has been used to assess the postural stability in the framework of the uncontrolled manifold hypothesis, which assumes that the CNS acts in a space of control variables selecting a value or a time profile of a performance variable that needs to be stabilized. Therefore, the CNS is able to control the stability of the center of pressure (performance variable), that shifts in the anterior-posterior direction, manipulating the M-modes index (control variable) (Danna-Dos-Santos et al., 2007; Falaki et al., 2017a). This approach defines M-modes as eigenvectors in the space of muscle activations using the principal component analysis and then investigates the variance in the M-mode space quantifying two components of inter-trial variance: the variance component within the UCM space (VUCM) that has no effect on the performance variable and the variance component within the space orthogonal to the UCM (VORT) that influences the performance variable (Falaki et al., 2017a).

Thus, the two synergy models agree with the hypothesis that spinal, supraspinal, and afferent signals flexibly combine a few muscle synergies to generate a variety of muscle patterns (D’Avella and Bizzi, 2005) with the aim to extract muscle synergies in order to reduce the degrees of freedom of the musculoskeletal apparatus that provide great flexibility. However, the first synergy approach addresses a plausible solution to the redundancy problem, providing a credible and parsimonious description of how modular control may rely on a limited set of available synergies. On the contrary, the latter considers abundance not as a “problem” to be solved, but rather as a resource to exploit to stabilize variables that are typical for achieving the performance in a specific task.

#### 4.1.2 Synergy algorithms: NMF vs. PCA and the opportunity for new models

In the literature, different models have been developed to decompose the EMG signals into muscle synergies to analyze human motor control (Brambilla et al., 2023a). The existing models are spatial or synchronous synergies (Tresch et al., 1999; Cheung et al., 2005), invariant temporal components or temporal synergies (Ivanenko et al., 2004) and spatiotemporal or time-varying synergies models (D’Avella et al., 2003; D’Avella et al., 2006). Both the spatial and temporal synergies are invariant synergy models because they rely on time-invariant modules: in spatial synergies, invariant muscle weights are modulated by variant temporal coefficients; in temporal synergies, temporal invariant synergies modulate variant muscle weights (Brambilla et al., 2023a). Instead, spatiotemporal synergies constitute a time-varying model because it is based on a collection of scaled and shifted waveforms, each one of them specific for a muscle or channel; in other words, the model can capture consistent relationships between the muscle activation waveforms across different muscles and over time. The time-varying model provides a more parsimonious representation of the muscle activity compared to the invariant model; however, some studies have shown evidence that muscle synergies are synchronized in time, therefore the invariant model is the most frequently applied method for the synergy extraction (Ebied et al., 2018). Both temporal and spatial models extract muscle synergies by applying a matrix factorization technique. The most common factorization algorithms to extract synergies for myoelectric control and clinical purposes are PCA and NMF. In particular, PCA was used in all the studies where postural control was investigated with the UCM method (e.g., Falaki et al., 2023; Freitas et al., 2020), while NMF was used to extract muscle synergies during gait analysis or reaching movements (e.g., Bai et al., 2021; Rodriguez et al., 2013). PCA uses the muscle activation matrix covariance to identify components that best describe the variance of the input data while minimizing the covariance of the basis vectors, while the NMF algorithm relies on a cost function to quantify the quality of approximation of the data matrix M (obtained by EMG signal processed) and it factorizes non-negative matrices W (synergy weights) and C (activation coefficients) where M ≈ WC and the values of W and C are updated and optimized to find the local minima numerically (Ebied et al., 2018). Both extraction algorithms share the same model and have comparable performance on both EMG and joint motion data, but each one imposes different constraints on the input signals and extracted synergies (Zhao et al., 2022a). PCA constrains the matrix of synergy vectors (W) to be orthogonal; the first component has the largest variance and the variance progressively decreases for each component (Ebied et al., 2018). Interestingly, this feature does not seem to resemble any specific feature of the motor control system. Furthermore, the data input can be negative and they are from Gaussian distributions. On the contrary, NMF can be used for both Gaussian and non-Gaussian data, both input and output are constrained to be non-negative and the extracted synergies are tied to be independent. In this way, PCA can be considered a very versatile method and adaptable to negative data (such as kinematics) but probably it is not the best algorithm to describe neural control at the muscle level because the components yielded using PCA impose a constraint that does not reflect the physiological organization. Imposing the non-negativity of the extracted synergies, NMF is particularly appropriate for clinical explanations because it reflects the non-negative nature of neural commands and muscle contractions. Moreover, EMG signals are usually non-Gaussian data and have a lot of non-linear and non-stationary components, which makes the NMF more performant than PCA in muscle synergy extraction (Zhao et al., 2022a). Although NMF can reconstruct the original EMG signals with a proper number of synergies, it cannot represent non-linear relations between muscles in the extracted synergies, such as the agonist-antagonist relationships. However, recent developments are considering also non-linear models and models that include the task space variables (e.g.,: kinematics) into synergistic models. Indeed, the autoencoder and the mixed-matrix factorization (MMF) have been developed. The autoencoder is a non-linear algorithm that has been developed to extract non-linear coupling information among muscles (Zhao et al., 2022a), while the MMF algorithm is a gradient descent algorithm for extracting synergies from a data matrix with a mixture of unconstrained and non-negative components allowing to obtain synergy vector W that can be positive, negative or zero and this allows the algorithm to also take kinematic data into account (Scano et al., 2022). These new algorithms aim at combining the study of muscle synergies and kinematics to explain the connection between the activation of muscles at the neural level with the actual execution of movement. Moreover, it was observed that muscle patterns are characterized by the presence of phasic and tonic components in the EMG signals, where the phasic components are related to accelerating and decelerating the joints, while the tonic components are responsible for balancing gravity and stabilizing the movement in presence of perturbations (Brambilla et al., 2023b). The NMF algorithm, is not able to identify the phasic and tonic synergies, but it leads to the extraction of “hybrid” synergies that incorporate a mixture of phasic and tonic components, and so, MMF can still be applied, because it allows to separate the two EMG components, because it can factorize the negative phasic components, which NMF cannot use. In this way, the role of the negative components of the signal can be interpreted and improve the muscle synergy analysis.

#### 4.1.3 Extending the sample of patients to generalize results

All the studies analyzed PD patients which are in the early stages of the disease, with a mild degree of disability. In fact, the majority of investigated PD patients are classified from the I to III stage of the Parkinson’s disease according to the Hoehn and Yahir scale (e.g., Allen et al., 2017; Falaki et al., 2017a; Falaki et al., 2023). At this level of disability, the Parkinson’s symptoms are not major: patients have unilateral or bilateral involvement without impairment of balance and only in the worst cases analyzed, they are affected by a mild to moderate disability with impaired postural reflexes, but in any case, they maintain their physical independence. Patients with higher disability are not presented in any of the reviewed studies, probably because it is more difficult to evaluate motor symptoms of such patients since, with the disease’s progression, the degeneration and disruption of neurons in substantia nigra increase, worsening motor and non-motor symptoms that are non-responsive to levodopa so PD patients cannot perform tasks in order to extract motor synergies (Coelho and Ferreira, 2012). This lack of evaluation does not allow to assess how muscle synergies change in the later stages of the disease. This assessment would be meaningful to monitor the long-term use of levodopa that may lead to a complication of motor functions causing fluctuations and dyskinesias, so a muscle synergy assessment in later stage would allow to evaluate how PD patients’ motor functions degenerate and how clinical research can step in to reduce this deterioration (Radhakrishnan and Goyal, 2018). When the studies compare PD patients with healthy subjects, they usually analyzed on average 20 subjects split in half between patients and healthy controls; while when they assess only patients with Parkinson’s disease, the studies assess a number of subjects ranging from 3 to 10 subjects. These numbers are not high enough to draw generalized conclusions useful to fully understand the pathophysiology of Parkinson’s disease (Zhao et al., 2023). Indeed, between the different studies, we struggle to find common findings that would allow us to deduce general findings for all subjects with PD. In recent years, there have been studies in which the number of subjects evaluated has risen to around 20 for both control and Parkinson’s cases (Thenaisie et al., 2022; Ghislieri et al., 2023). Despite that, more can be done to generalize the results and obtain significant evidence which, by contrast, is already available in the case of patients with stroke (Zhao et al., 2023).

#### 4.1.4 The need of homogeneous pipelines

The results summarized in this review are sometimes difficult to compare because EMG processing methods are variable between the several studies. Indeed, the choice of different EMG pre-processing techniques, such as the application of different low-pass filters, could alter the data information content available to the factorization algorithm modifying the weights and activation coefficients of the synergies extracted. Some studies noted that applying low-pass filter with a different cut-off frequency, the dimensionality of the extracted synergies might change, as well as the weights of the extracted synergies. In fact, it was observed that if the cut-off frequency of the low-pass filter is higher, the weights of the dominant muscles contributing to the synergies are lower, so higher low-pass cut-offs reduce the importance of the dominant muscles in explaining the variance of the original data. On the contrary, particularly low-pass cut-offs increase the relative amplitude of the active muscles with respect to the inactive ones thus decreasing the signal-to-noise ratio of the weight coefficients (Kieliba et al., 2018). In agreement with this observation, considering the lower-limb muscle synergies, the use of a higher low-pass cut-off results in lower VAF and higher synergies variability. So, lower-limb synergies weight coefficients and the VAF criterion are sensitive to the low-pass cut-off frequency (Shuman et al., 2017). The VAF is also influenced by the stacking data techniques which can be different from the studies. The studies organized the EMG data following three different methods: the concatenation method, that concatenates all trials of a subject together to form a muscle activation matrix for synergy extraction (e.g., Freitas et al., 2020; Bai et al., 2021; Ghislieri et al., 2023); the averaging method, that requires to average the muscle activations from *n* trials obtaining a muscle activation matrix from each subject (such as in Mileti et al., 2020a; Hu Z. et al., 2019) and the single trial method in which the study extract muscle synergies from each trial, as we observed in Rodriguez et al. (2013), Roemmich et al. (2014). Depending on the stacking method used, it was shown that the calculated VAF changes and specifically using the averaging method it was significantly higher than applying concatenation and trial-by-trial methods, while these last two methods did not show any statistical difference (Zhao et al., 2022b). Additionally, in the screened studies often the stacking data method is not specified, so it is not clear how the EMG data are organized in the matrix used to extract muscle synergies and this also limits analysis and comparison between the studies. For these reasons, it is important to standardize the EMG pre-processing methods so as to obtain results which are comparable and that allow to define common pathological features of Parkinson’s subjects analyzed in the different studies. In conclusion, the community should develop uniform and standardized methods, that are not yet available, in order to create repeatable and robust pipelines that are minimally affected by intra/inter-operator variability and allow to reach solid and generalized conclusions useful in better understanding how to intervene to better manage the course of the disease.

### 4.2 Impact on the clinical practice

This section reports the possible lines of research for improving the usability and the impact of muscle synergies in specific research and clinical topics related to PD.

#### 4.2.1 Characterization of synergistic control in Parkinson’s patients

It is well-known that Parkinson’s disease is a progressive disorder associated with changes in brain structures which result in a reduction in the complexity of the motor control system causing an impaired motor performance (Falaki et al., 2023; Ghislieri et al., 2023). As a consequence, the motor control system in subjects with Parkinson’s disease is in general characterized by a lower number of muscle synergies compared to age-matched healthy subjects to control the same movements. Indeed, in PD patients, some of the biomechanical functions identified in the control of movements are merged into a single function, and specifically, muscle synergies related to body stabilization and dynamic postural control appear to be the most affected by this muscle synergy merging, suggesting a reduced ability to independently control muscle synergies deputed to those biomechanical functions (Ghislieri et al., 2023). With MMF, this merging might be investigated thoroughly by attributing biomechanical functionality to each extracted synergy, thus observing whether the impaired movement is related to an incorrect recruitment of muscles or an abnormal coupling between muscles and biomechanics. Proving that PD patients require a lower number of muscle synergies to reach good model reconstruction compared to healthy adults, synergies and ASAs indices could be considered as biomarkers to control the disease’s progression, and the effectiveness of followed therapy (Falaki et al., 2023). Additionally, it would be also important to compare indices from synergistic assessment with a broader range of clinical indices reflecting postural stability and freezing of gait to understand the association between the clinical analysis and muscle synergies analysis between the two fields to find some rehabilitation therapies that would be more effective (Falaki et al., 2016). Such features could be investigated in the light of different synergistic models, involving not only spatial methods but also temporal. Furthermore, since the number of muscle synergies extracted was always 4 or 5 in all the analyzed studies and this number typically did not change after drug assumption, Allen et al. demonstrated that the number of motor modules recruited for a motor task is not an appropriate metric to identify changes in neuromuscular control to evaluate the improvements in motor performance with rehabilitation. Indeed, the number of motor modules did not increase in subjects with Parkinson’s disease, despite they showed clinically meaningful improvements in measures of balance control, gait, and disease symptoms. On the contrary, Allen et al. discovered that changes in the distinctness, coactivity, and generalization of motor modules highlight an improvement of basal ganglia function. The distinctness of motor modules defines the differences between motor modules’ structure that allow a better motor performance; motor module coactivation reflects the simultaneous activity of anatomically similar muscles (e.g., ankle plantar-flexors) and/or coactivation of muscles crossing different joints and the generalization of motor modules results in a major sharing of motor modules across the tasks performed which reflects the presence of common substrates at neural level.

#### 4.2.2 Temporal analysis of muscle synergies during Parkinson’s progression

Since the development of PD progressively alters motor control, longitudinal assessments that monitor the course of the disease may provide insights into the progress of the motor capability and the effects of therapy. Longitudinal studies still assess the stages of Parkinson’s disease mostly using clinical scales such as the Hoehn and Yahir scale. However, these clinical scales show a poor resolution in evaluating motor capabilities and potential inter-operator biases, so they cannot analyze in detail how muscle control changes as the disease progresses or during rehabilitation therapy. Muscle synergy-based approaches improve such assessments with a neural analysis directly connected to the source of the disease and can observe how muscle synergies change in time becoming more similar to muscle synergies of healthy subjects after rehabilitation training. In this regard, it is necessary to create reference datasets obtained extracting muscle synergies from healthy subjects in order to compare these data with the synergies extracted from Parkinson’s patients before and after a rehabilitation period in order to investigate if there is an alignment toward physiological synergies. These types of studies are needed because they allow to understand if the rehabilitation therapy has produced beneficial and therapeutic effects at the neural level. Unfortunately, they are very limited in number: most of the available literature is based on single-session studies which cannot show a disease improvement due to therapy. Two studies only led longitudinal studies investigating how the conditions of PD patients changed after therapy: Ghislieri et al. demonstrated that PD patients improved smoothness and decreased the variability of neural commands during the motor task after DBS surgery (Ghislieri et al., 2023); Allen et al. compared PD patients before and after a 3-week rehabilitation therapy observing that participants decreased motor module variability and increased motor module distinctness and consistency in both walking and reactive balance (Allen et al., 2017). Furthermore, studying how muscle synergies change over time could reveal new insights into the deterioration of motor abilities. This could include the longitudinal monitoring of muscle synergies in patients at different stages of the disease to understand how the motor system attempts to compensate for motor losses in the early stages versus advanced stages. Indeed, several motor compensatory strategies are adopted to help movements, such as changing the walking rhythm or shifting the weight while stepping (Nonnekes et al., 2019). Motor losses have been investigated at the cortical level, observing how brain connectivity changes with dopamine loss; however, muscle synergies may add information on how these mechanisms reflect on neural commands and the biomechanical response (Passaretti et al., 2024).

#### 4.2.3 Impact of pharmacological therapy and deep brain stimulation (DBS) on muscle synergies

The effects of drugs like levodopa or interventions like DBS have been studied in terms of improving motor abilities, but their specific effect on muscle synergies has not yet been fully explored. Indeed, dopaminergic treatments have been demonstrated to improve gait speed, step length, and also trunk and arm movements during gait; therefore, it could be interesting to examine how muscle synergies change before and after levodopa administration. Some studies assessed patients in OFF-drug and ON-drug conditions, observing that the number of extracted synergies did not change between conditions (Mileti et al., 2020a; Roemmich et al., 2014). However, Falaki et al., showed that dopaminergic-replacement drugs lead to higher indices of multi-muscle synergies and higher ASA indices remarking the role of the basal ganglia circuitry for the control of postural stability and gait initiation (Falaki et al., 2017b). Moreover, it was noted that there are some differential effects between the first-dose and chronic PD medications on synergy metrics and this suggests that long-term exposure to dopamine-replacement medications may modify neuronal circuits involved in synergic control of movements (Falaki et al., 2018). Muscle synergy analysis would be a valuable tool for evaluating changes at the neural level during the treatments; however, only single-session studies were conducted, and, therefore, the effects of long-term dopaminergic treatments on muscle synergies were not assessed in the literature. Moreover, since long-term use of Levodopa may lead to toxicity causing new motor problems (dyskinesia) (Radhakrishnan and Goyal, 2018), some alternative dopaminergic replacement drugs may be developed to prolong the effectiveness of the medication so that patients with Parkinson can continue to modify neuronal circuits to try to improve their motor control avoiding toxic effects. It would be interesting to observe these improvements through muscle synergies extraction conducting a longitudinal follow-up study to analyze the multi-muscle synergies and ASAs indices across H&Y stages to evaluate the predictive value of these indices.

An alternative treatment is the installation of a DBS implant, that has been demonstrated to be an effective treatment for advanced PD patients, leading to good control of the PD symptoms, like rigidity, bradykinesia, tremor, and motor fluctuations. Muscle synergy analysis offers a valuable instrument for assessing motor control changes induced by DBS. However, only few studies assessed the effects of DBS with synergies, showing that patients subjected to DBS did not change the number of synergies that remained lower than healthy controls; however, the neuromuscular robustness of PD patients increased after DBS, becoming not distinguishable from that of controls. This result documented an improvement in smoothness and a decrease in the variability of neural commands used during the motor tasks (Ghislieri et al., 2023). DBS also influenced the ASAs initiations, decreasing the difference in ASAs timing with respect to controls (Falaki et al., 2018). A more detailed assessment of the effects of DBS on motor control through synergy analysis in long-term treatments may give insights into motor control reorganization. Finally, analyzing how muscle synergies change in response to different stimulating levels of DBS can be used for optimizing the DBS parameters in order to personalize the therapy and maximize the efficacy, similarly to the DBS parameter programming based on fMRI patterns (Boutet et al., 2021).

During the rehabilitation of PD patients, functional electrical stimulation (FES) is an effective and non-invasive method for tremor suppression (Meng et al., 2022). Moreover, this method has been shown to be effective also for improving gait and reducing bradykinesia (Popa and Taylor, 2015). However, no studies assessed the effects of FES on motor control with muscle synergies. This method may provide insights into how motor control and muscle coordination change when FES is applied.

#### 4.2.4 Study of muscle synergies in tremor and rigidity

Rigidity and tremor are hallmark symptoms of Parkinson’s disease, but how they influence muscle synergies is not well understood. Tremor showed to be related to inter and intra-muscular synchronization. Indeed, muscles shared the same firing frequency during tremor and the intensity of tremor was correlated to the degree of inter-muscular synchronization and the number of synchronized muscles (He et al., 2015). Exploring how muscle synergies are involved in maintaining and modulating Parkinsonian tremor could clarify the mechanisms underlying this symptom and offer new intervention strategies. Hu et al. showed that muscle synergies of tremor displayed two bursting components in time profiles, corresponding to the alternating drives to antagonistic muscles, and, when the tremor is inhibited by cutaneous stimulation, synergy analysis demonstrated that the inhibition of tremor takes place in the spinal motoneurons (Hu Z. et al., 2019). Since the spinal circuitry is directly involved in tremor and its inhibition, muscle synergies may play a fundamental role for a deeper understanding of this phenomenon.

Another symptom that can interfere with daily activities is rigidity, that affects both posture and movements. The pathophysiological mechanisms that are responsible for rigidity still remain unclear and rigidity may be associated with the co-contraction of muscles around joints in response to postural perturbations (Park et al., 2015) and may result from overly synchronized or poorly coordinated muscle synergies in which many muscles are activated at the same time. Muscle synergy analysis could provide insight into the mechanisms at the neural level that cause rigidity and could reveal how the CNS overcompensates with exaggerated muscle co-activation and offer new therapeutic approaches.

#### 4.2.5 Muscle synergies in locomotion and posture

Parkinson’s disease significantly affects posture and gait (slow walking, small steps, difficulty starting and stopping). Postural instability is one of the symptoms of PD and it is related to a higher risk of falls. The postural instability is an impaired neural control of posture and it was assessed through multi-muscle synergy indices that can be more suitable with respect to muscle synergies (Freitas et al., 2020). Investigating synergies involved in maintaining posture, ASA, APA, and balance, could be useful in better understanding the mechanisms that lead to frequent falls in Parkinson’s patients and in developing targeted rehabilitation exercises. Posture also affects gait ability. Instrumented gait analysis already widely proved its validity in providing an objective assessment of the functional performance of PD patients based on kinetic, kinematic, and spatiotemporal gait parameters, but few studies addressed motor coordination during locomotion (Di Biase et al., 2020). However, the alteration of gait parameters could be the result of neural abnormalities in which different muscle coordination patterns are altered. Studies assessed muscle synergies during gait and found a merging of biomechanical functions, suggesting a reduction of the ability of the CNS to control independently muscle synergies (Ghislieri et al., 2023). Muscle synergies in PD patients during gait have been assessed with the spatial synergy model; however, given the repetitive nature of this task, the temporal model may be more appropriate and give more insights into the coordination and the temporization of muscle activations. Studying muscle synergies during walking could help identify abnormal patterns and potential motor compensations in Parkinsonian gait, especially in situations of “freezing of gait” (episodes where patients suddenly freeze). The origin of this phenomenon is not well understood, but some studies related the freezing event to altered coordination of the gastrocnemii and tibialis anterior muscles before freezing, affecting both the timing and the magnitude of EMG activity (Nieuwboer et al., 2004). Therefore, muscle synergies may provide an additional instrument for understanding this phenomenon and finding therapeutic approaches to reduce its manifestation.

#### 4.2.6 Muscle synergies in the context of the upper limbs and of whole-body control

While much research focuses on locomotion and posture, muscle synergies in the upper limbs are less studied. Analyzing how Parkinson’s patients use muscle synergies in their hands and arms during fine motor tasks (such as grasping objects or writing) could improve understanding of motor difficulties in the upper limbs, which significantly impact the quality of life. For this kind of movements, the link between muscle activity and biomechanical output may be fundamental, and, therefore, kinematic-muscle synergies are the appropriate method for assessing upper-limb and hand motor control. Moreover, in our screening we found studies that analyzed movements of the upper limb (e.g., Hu Z. et al., 2019) and studies that evaluated the lower limb during the gait or postural control (e.g., Allen et al., 2017), but no study investigated whole-body movements. In subjects affected by Parkinson’s disease, it is possible to observe a reduction of supplementary motor area (SMA) activity due to the degeneration of dopaminergic cells within the substantia nigra pars compacta and this is associated with gait deficits involving both upper and lower limbs. Gait impairment can be investigated also by assessing arm swing because this upper limb movement could drive and shape the leg muscle activity. Indeed, some studies demonstrated that arm swing is integrated into locomotion via tight interlimb coordination in healthy gait (Weersink et al., 2022). So, combining the gait analysis with the assessment of the upper limbs, we can investigate better how subjects with Parkinson’s disease, which are characterized by a reduced and more asymmetric arm swing, move around the place and how they control their position and their movements compared to healthy subjects. In particular, extracting muscle synergies both from upper limb and lower limb muscles, the changes in the interlimb coordination in PD patients during motor control and the effects of the synergistic control of the upper limb on the muscles leg activation can be investigated, having a framework more complete of the pathology symptoms to understand how we can intervene from a rehabilitation point of view.

#### 4.2.7 Personalization of motor rehabilitation

Rehabilitative therapies for Parkinson’s patients are often generic and may not always consider specific muscle alterations of each patient. Indeed, each patient may manifest different and specific impairments of motor control. Monitoring muscle synergies could allow for the design of personalized rehabilitation interventions aimed at restoring or optimizing altered synergies, rather than applying standardized approaches.

So far, muscle synergy analysis in PD patients has only been used to implement synergistic approaches for evaluation without applying the extracted results as a starting point to create customized therapies. Indeed, most of the studies use only an evaluation approach in order to observe what were the differences between PD and healthy people in terms of movement control and gait performance (Falaki et al., 2017a; Falaki et al., 2016; Falaki et al., 2023; Rodriguez et al., 2013), or if there were some improvements when PD individuals followed dopaminergic therapy or underwent DBS (Mileti et al., 2020a; Roemmich et al., 2014). However, they did not apply this information to define some specific therapies based on the level of disease progression or based on specific synergistic characteristics of the subjects with PD. On the contrary, the synergistic approaches used to point out the understanding of the mechanisms of motor control in healthy people might be used to create specific rehabilitation exercises to train muscle synergy-inspired motor functions in PD patients in order to improve the motor activity in individuals with PD. In this way, it would be possible to customize therapies and interventions, for example, to train or promote the recruitment of specific synergies. This approach was partially implemented in pilot studies regarding post-stroke rehabilitation, where muscle synergies were used to create patterns for Functional electrical stimulation or for robot-mediated treatment (Tropea et al., 2013; Niu et al., 2019). A similar rehabilitation therapy might be implemented in medical stimulation devices using patterns based on muscle synergies extracted by healthy subjects in order to stimulate lower limb muscles of individuals with Parkinson’s disease to try to improve their muscular activity during gait tasks and to reduce bradykinesia. Alternatively, some studies have already demonstrated that some physical exercises may be beneficial for people affected by Parkinson’s disease and over the last decade they became essential elements in the treatment plan alongside the pharmacological therapy (Petzinger et al., 2010; Almikhlafi, 2023). Moreover, future rehabilitation therapies for PD patients may be focused on improving the structure of motor modules in terms of coactivity, generalization, and distinctness, instead of proving to increase the number of synergies extracted so that may there be an improvement in patients’ motor performance that can be demonstrated clinically (Allen et al., 2017).

Technologies such as biofeedback or robotics could be used to redirect inefficient muscle synergies, teaching patients how to improve motor control by visualizing muscle synergies in real-time during exercise.

#### 4.2.8 Impact of fatigue and automatic motor control

Fatigability is a less explored symptom in Parkinson’s disease, but it is extremely common. Indeed, fatigue is a frequent, independent non-motor symptom in PD appearing early and persisting throughout the disease course, and it is difficult to establish uniform diagnostic criteria for PD-related fatigue, since several non-motor symptoms appear to be associated with fatigue (Siciliano et al., 2018). Studying muscle synergies during tasks requiring prolonged effort or in fatigue conditions could reveal important information, clarifying the mechanisms through which fatigue worsens motor performance in Parkinson’s patients.

Moreover, Parkinson’s disease also affects the shift from automatic movements to voluntary movements.

Neuroimaging studies revealed that impaired motor automaticity in PD is related to less efficient neural coding of movement, instability of the automatic mode within the striatum, and use of attentional control and/or compensatory efforts to execute movements usually performed automatically in healthy people (Wu et al., 2015). Studying synergies in the shift from automatic to voluntary movements could help better understanding of how the disease impacts on movements that should normally be effortless. For instance, recruiting motor modules that are more distinct in structure may result in producing well-defined biomechanical output; an increase in motor module coactivation reduces the sparsity of muscle representation within a module leading to an improvement in the generation of specific biomechanical outputs and makes the movement more energy-efficient; and finally, the increase of generalization of motor modules across walking and reactive balance, increasing the sharing of common sets of motor modules, could indicate an improvement in automatic control of gait (Allen et al., 2017).

#### 4.2.9 Interaction between muscle synergies and cognitive functions

Parkinson’s disease is not just a movement disorder, but it also affects cognitive function (especially motor planning and multitasking). Exploring how muscle synergies are influenced by simultaneous cognitive tasks could reveal new connections between motor and cognitive systems. Multiple factors, such as specificity, intensity, frequency, difficulty, and complexity, can contribute to the efficacy of these exercises and they are often designed to incorporate motor and cognitive functions through dual-tasking. The rehabilitative exercises that to date are typically used and seem to bring satisfactory results are treadmill exercise, Tai chi, tango dancing (e.g., Allen et al., 2017), boxing, and cycling, and these can be accomplished with advanced technology, such as virtual reality (VR) using a computerized simulation to implement also some exercises that can develop cognitive tasks. Some studies observed that treadmill exercise has a positive impact on stride time and swing time variability, leading to a more stable gait rhythm and, also, results in an increase of the levels of dopamine 2 receptor (D2) in the caudal basal ganglia contributing to affect neuroplasticity and inducing modification of the indirect pathway of automaticity, deriving an abnormal inhibitory effect. Moreover, exercises, such as dancing and treadmill exercise could prevent declining of cognitive function and may be able to improve it (Almikhlafi, 2023). Hence, future aims would be to define some rehabilitation treatments that provide for combining physical and cognitive exercises to intervene simultaneously on motor and non-motor symptoms and try to improve basal ganglia function in order to have a more effective modular organization of muscle activation that affects neurological coordination ability defining the PD patients walking ability. Studying how muscle synergies change when patients must perform both a motor task and a cognitive task (such as walking and talking) could provide useful insights into how the disease affects the simultaneous control of actions.

#### 4.2.10 Integrating muscle synergy analysis into animal models

Researches in the literature suggest that PD results from a complex interaction between environmental and genetic risk factors, but the etiology and most PD causes are still unknown and so only symptomatic therapies such as pharmacotherapy, neurosurgery, and physiotherapy are available. Therefore, clinical needs new tools to understand the pathophysiology of PD in order to discover new strategies to prevent, stop, or slow disease progression (Kin et al., 2019). Muscle synergies offer a complementary point of view of the PD to the animal models that are used for assessing the pathophysiology of the disease and the effects of treatments. While muscle synergies provide a quantitative measure of motor control, animal models investigate the pathophysiology of the neural mechanisms. The methods used in animal models can be categorized into two main groups: the neurotoxin-based model, developed by introducing some neurotoxins that induce the rapid degeneration of nigrostriatal dopaminergic neurons; and the genetic model, obtained by manipulating specific genes to reproduce the features of the disease and understand the molecular mechanisms. Both these models have limitations, such as the lack of formation of Lewis body, the difference with the human conditions and the failure of inducing significant loss of dopaminergic neurons (Chia et al., 2020). However, animal models provide good information to study the pathophysiology of PD and to find new therapies (Hattori and Sato, 2007). Indeed, animal models have shown that nigrostriatal dopaminergic degeneration directly correlates with motor deficits like bradykinesia, rigidity, and tremors, contributing to reduced motor output and impaired movement initiation. Furthermore, animal models provide detailed analyses of motor deficits, such as reduced locomotor activity, impaired gait, and altered postural control, and allow to observe the progression of motor and non-motor symptoms, providing insights into disease stages (Thirugnanam and Santhakumar, 2022). All these aspects are fundamental for the development of new therapies to slow the progression of the disease. Future research may incorporate muscle synergy analysis into animal models in order to gain a better understanding of motor symptoms caused by specific pathophysiological factors associated with Parkinson’s disease. Indeed, muscle synergies represent an approach to investigate the principles of neuromuscular control during motor performance, providing a better insight than kinematics only (Safavynia et al., 2011). Therefore, by associating animal models and muscle synergy analysis, future studies may discover new strategies specific for each case of PD and find biomarkers for an early detection of the disease. Finally, the integration of muscle synergies into animal models may help the translation between the animal models and the clinical practice for both the understanding of the pathology and the effects of the therapy.

## 5 Conclusion

Many studies found in the literature deal with muscle synergy analysis, but there are still several challenges ahead for a systematic application of synergy approaches to PD patients. Firstly, the number of subjects enrolled is not sufficiently high to reach standardized conclusions which can be summarized for all patients with Parkinson’s disease in order to define the pathophysiology features and to obtain reliable results regarding the effectiveness of both pharmacological and rehabilitation therapies. Another problem which limits the comprehensive view on how to improve the study and treatment for this disease is the lack of guidelines and uniformity for pre-processing EMG data in order to compare the results obtained from different studies. In this way, the scientific community may compare the results of the same therapies applied from different studies to understand if they are similar and repeatable for all patients with Parkinson’s disease or they may compare PD patients’ characteristics by analyzing the subjects in several conditions to assess how they face different challenges and if the disease’s symptoms change or are affected by the environment in which they are analyzed. Muscle synergy-based approaches improve such assessments with a neural analysis; however, the most important obstacle to overcome is that all the results found in the papers screened are still mainly for assessment and are not applied by clinics to improve rehabilitation and medical care but only for evaluation. Hence, in future studies, the number of subjects analyzed should increase in order to reach firm conclusions and a standardized pipeline should be defined in order to create a starting data set that can give comparable results across studies. Moreover, the community should find a way of using muscle synergies analysis to evaluate the characteristics of PD patients in order to assess how motor control in PD patients works. Finally, it is needed a major link between the muscle activation and the kinematic movements associated in order to do a task space analysis to understand better how the kinematics variables used to perform motor tasks are explained at the neural level.

## Data Availability

The original contributions presented in the study are included in the article, further inquiries can be directed to the corresponding author.
